# Arsenic Response of Three Altiplanic *Exiguobacterium* Strains With Different Tolerance Levels Against the Metalloid Species: A Proteomics Study

**DOI:** 10.3389/fmicb.2019.02161

**Published:** 2019-09-26

**Authors:** Juan Castro-Severyn, Coral Pardo-Esté, Yoelvis Sulbaran, Carolina Cabezas, Valentina Gariazzo, Alan Briones, Naiyulin Morales, Martial Séveno, Mathilde Decourcelle, Nicolas Salvetat, Francisco Remonsellez, Eduardo Castro-Nallar, Franck Molina, Laurence Molina, Claudia P. Saavedra

**Affiliations:** ^1^Laboratorio de Microbiología Molecular, Departamento de Ciencias Biológicas, Facultad de Ciencias de la Vida, Universidad Andrés Bello, Santiago, Chile; ^2^Center for Bioinformatics and Integrative Biology, Facultad de Ciencias de la Vida, Universidad Andrés Bello, Santiago, Chile; ^3^BioCampus Montpellier, CNRS, INSERM, Université de Montpellier, Montpellier, France; ^4^Sys2Diag, UMR9005 CNRS ALCEDIAG, Montpellier, France; ^5^Laboratorio de Microbiología Aplicada y Extremófilos, Departamento de Ingeniería Química, Facultad de Ingeniería y Ciencias Geológicas, Universidad Católica del Norte, Antofagasta, Chile; ^6^Centro de Investigación Tecnológica del Agua en el Desierto (CEITSAZA), Universidad Católica del Norte, Antofagasta, Chile

**Keywords:** *Exiguobacterium*, arsenic, tolerance, proteomic, polyextremophile

## Abstract

*Exiguobacterium* is a polyextremophile bacterial genus with a physiology that allows it to develop in different adverse environments. The Salar de Huasco is one of these environments due to its altitude, atmospheric pressure, solar radiation, temperature variations, pH, salinity, and the presence of toxic compounds such as arsenic. However, the physiological and/or molecular mechanisms that enable them to prosper in these environments have not yet been described. Our research group has isolated several strains of *Exiguobacterium* genus from different sites of Salar de Huasco, which show different resistance levels to As(III) and As(V). In this work, we compare the protein expression patterns of the three strains in response to arsenic by a proteomic approach; strains were grown in absence of the metalloid and in presence of As(III) and As(V) sublethal concentrations and the protein separation was carried out in 2D electrophoresis gels (2D-GE). In total, 999 spots were detected, between 77 and 173 of which showed significant changes for As(III) among the three strains, and between 90 and 143 for As(V), respectively, compared to the corresponding control condition. Twenty-seven of those were identified by mass spectrometry (MS). Among these identified proteins, the ArsA [ATPase from the As(III) efflux pump] was found to be up-regulated in response to both arsenic conditions in the three strains, as well as the Co-enzyme A disulfide reductase (Cdr) in the two more resistant strains. Interestingly, in this genus the gene that codifies for Cdr is found within the genic context of the *ars* operon. We suggest that this protein could be restoring antioxidants molecules, necessary for the As(V) reduction. Additionally, among the proteins that change their expression against As, we found several with functions relevant to stress response, e.g., Hpf, LuxS, GLpX, GlnE, and Fur. This study allowed us to shed light into the physiology necessary for these bacteria to be able to tolerate the toxicity and stress generated by the presence of arsenic in their niche.

## Introduction

Arsenic is a ubiquitous and abundant metalloid, frequently found in water bodies, accumulated in soil or as particulates in the air. There are two main oxidation states found in the environment: +5 [arsenate, As(V)] and +3 [arsenite, As(III)] (Mandal and Suzuki, 2002; Xiao et al., 2016). As(V) can interfere with important cell processes causing the substitution of PO_4_^–2^ inside the cell (Goyer and Clarkson, 2001; Andres and Bertin, 2016). On the other hand, As(III) is more toxic because it reacts with thiol groups, oxidizing cysteine residues inhibiting enzyme function, additionally it interferes with the maintenance of the redox state reacting with anti-oxidative molecules (Liu et al., 2001; Slyemi and Bonnefoy, 2012). Thus, cellular damage caused by arsenic is associated with its chemical nature, the generation of Reactive Oxygen Species (ROS) (Bhattacharjee and Rosen, 2007; Jomova et al., 2011), and the activation of the ROS-detoxifying machinery (Hrimpeng et al., 2006).

The presence of arsenic in its natural form occurs form geologic events (Bissen and Frimmel, 2003; Follmann and Brownson, 2009). Additionally, anthropogenic activities, like the mining industry, are the major causes of contamination and related health problems (Pacyna and Pacyna, 2001; Han et al., 2002). In northern Chile, the consumption of contaminated waters is the main cause of arsenic-related health problems (Smith et al., 1998; Flynn et al., 2002; Ferreccio and Sancha, 2006; Frazer, 2012). Specifically, in the Salar de Huasco there has been reports of toxic arsenic concentrations for humans with a temporal variation between 34 and 268 mg kg^–1^ in sediments (De Gregori et al., 2003; Herrera et al., 2009) and 70 mg L^–1^ in water (Herrera et al., 2014). However, the spatial variation in the concentration of the metalloid in the area is still relatively unknown.

There are four bacterial resistance mechanisms to arsenic described and the most studied are based on the toxicity reduction and expulsion of the arsenic species from the cell (Nies and Silver, 1995; Cánovas et al., 2003). The most common mechanism found in bacteria is the one mediated by the *ars* operon (Xiao et al., 2016). This operon mediates the reduction of As(V) to As(III) by the enzyme Arseniate reductase (ArsC) and the later expulsion from the cell trough the ArsB and/or ACR3 pumps (Slyemi and Bonnefoy, 2012). Another resistant mechanism is the dissimilatory reduction of As(V) through the genes in the *arr* operon, found mainly on bacteria grown in anoxic conditions, using this compound as the last electron acceptor (Malasarn et al., 2008; Ohtsuka et al., 2013). Additionally, successive methylations of As(III) by the arsenite methyltransferase ArsM for volatilization is another well-studied mechanism (Qin et al., 2006; Zhang et al., 2015). Finally, an oxidation-based mechanism to obtain energy while diminishing As(III) toxicity has been described in bacteria like *Alcaligenes faecalis* and *Thermus aquaticus* YT1 (Philips and Taylor, 1976; Gihring et al., 2001).

*Exiguobacterium* is a cosmopolitan genus composed by Gram-positive, facultative anaerobic, non-sporulating and motile rods that have been isolated from varied environments such as permafrost, salt lakes, deserts and even industrial wastes, suggesting a high plasticity, adaptation capacity, and tolerance to extreme environmental factors (Collins et al., 1983; Vishnivetskaya and Kathariou, 2005; Ordoñez et al., 2009; Vishnivetskaya et al., 2009). Strains from this genus have been reported to reduce and bio-accumulate arsenic (Anderson and Cook, 2004; Pandey and Bhatt, 2015), and show high resistance to oxidative stress (Takebe et al., 2007).

The *Exiguobacterium* sp. SH31 is a strain isolated from the chilean Altiplano area, in the Salar de Huasco sediments that has a high tolerance to As. The genome from this strain was sequenced and the genomic analyses revealed that it has a great variety of genes related to the response and tolerance to extreme environmental conditions (Castro-Severyn et al., 2017; Remonsellez et al., 2018). In regard to the arsenic resistance, it has the As(V) reduction *arsRDAXB* operon and also the *acr3* gene. The presence of *acr3* was reported for the first time in the *Exiguobacterium* genus in the genome of the S17 strain that was isolated from the Argentinian Altiplano (Belfiore et al., 2013), tolerates high concentrations of As(V) (150 mM) and As(III) (10 mM) (Ordoñez et al., 2013, 2015). Even though all *Exiguobacterium* strains have some version of the As(V) reduction operon, it is possible that the greater resistance presented by S17 and SH31 strains could be due the presence of ACR3 expulsion pump (Ordoñez et al., 2015). The presence of this gene has been previously reported in bacteria present on environment with high concentrations of arsenic (Cai et al., 2009).

The global molecular mechanisms in response to arsenic have been poorly described so far. Most of the studies are not comparable because they use different models, isolated in variable conditions and different As tolerance levels. Transcriptomic patterns of *Enterobacteriaceae* strain LSJC7 in response to As(V) and *Herminiimonas arsenicoxydans* in response to As(III) and As(V) revealed few constant elements, among these central metabolism and oxidative stress response (Cleiss-Arnold et al., 2010; Zhang et al., 2016). Comparative proteomic analyses of *Exiguobacterium* sp. S17 and *Klebsiella pneumoniae* exposed to arsenic have revealed that most of the proteins expression changes are related to general stress, oxidative stress, carbon metabolism and transport processes (Daware et al., 2012; Belfiore et al., 2013). These results show that the global response is not standard, and it does not depend exclusively on the described arsenic response systems, nevertheless, the level of arsenic tolerance is affected by the central metabolic response (Andres and Bertin, 2016). To better elucidate bacterial As resistance mechanisms at protein level, we aimed to study the stress response caused by the As(V) and As(III) in *Exiguobacterium* sp. For this, we compared the protein expression of three different strains of *Exiguobacterium* sp. isolated from different sites of the Salar de Huasco, where each of them had particular and different environmental conditions.

## Materials and Methods

### Bacterial Strains and Arsenic Tolerance

*Exiguobacterium* sp. SH31 was isolated from H4 site of the Salar de Huasco and its arsenic tolerance was described previously as [100 mM As(V) and 10 mM As(III)] (Castro-Severyn et al., 2017; Remonsellez et al., 2018). During February of 2017, another two *Exiguobacterium* strains SH0S7 and SH1S21 were isolated from sediment samples from H0 and H1 sites of the Salar de Huasco, respectively, (sites were described previously: Dorador et al., 2008). Strains were identified by 16S rRNA gene sequencing (ABI PRISM 3500xl Applied Biosystems – Centro de Secuenciación Automática de ADN, Pontificia Universidad Católica de Chile). Physicochemical parameters of sampling sites (temperature, salinity, pH) were recorded with a digital multi-parameter instrument (HI 98192 – HANNA Instruments) and total arsenic content was analyzed by IPC-MS (INQUISAL-CONICET, San Luis, Argentina). The arsenic tolerance of these two new strains was assessed by minimal inhibitory concentrations (MIC) assays. Briefly, bacterial cultures in Luria-Bertani broth (LB) were grown at 25°C with constant agitation (150 rpm) until 0.04 OD_600_. From this culture, we set up a micro plate with dilutions of As(III) and As(V) to a final concentration of 0.1–25 mM and 10–300 mM, respectively, for each strain. Finally, the plates were incubated at 25°C for 72 h with constant agitation, and OD_600_ was measured with a TECAN Infinite 200 PRO Nanoquant. Growth was monitored for 24 h at 25°C with OD_600_ measures every hour and curves were plotted using R package ggplot2 (Wickham, 2016).

### Growth Conditions and Protein Extraction

The three *Exiguobacterium* strains were grown in LB at 25°C with constant agitation (150 rpm) overnight and this culture was used as inoculum to three flasks for each strain: 50 ml of LB as control, 50 ml of LB-As(III), and 50 ml of LB-As(V). Flasks were cultured at 25°C, 150 rpm up to mid-exponential growth phase (OD_600_ 0.4). Arsenic conditions were particular to each strain, half of the MIC value was used as As(III) and As(V) treatment conditions. Bacterial cells were harvested by centrifugation (3,000 *g*, 10 min) and washed twice with tris–HCl buffer (50 mM, pH 8.5). Bacterial pellets were resuspended in 1 ml of the same buffer supplemented with 1 mM of PMSF (Phenylmethylsulfonyl Fluoride) and were sonicated at 40% amplitude (130 watts, 20 kHz) during 5 min (10 s on and 10 s off cycles) Ultrasonic Processor VCX-130, (Sonics, Inc.). The lysates were centrifuged at 24,000 *g* for 40 min at 4°C, the supernatants were recovered and stored at −80°C until use. Three independent assays for each strain and condition were performed. Protein concentrations were measured using the Coomassie (Bradford) Protein Assay (Thermo Fisher Scientific).

### Sample Preparation

Two-hundred μg of protein for each sample were lyophilized, resuspended in 200 μL of lysis buffer (8 M urea, 2 M thiourea, 40 mM tris, 4% w/v CHAPS, 65 mM dithioerythritol (DTE), protease inhibitor (Roche Diagnostics) and incubated at room temperature on a rotating wheel for 2 h. Next, the samples were centrifuged 30 min at 20,000 *g* and the supernatants were collected and stored at −80°C until use. Protein quantification was performed using the RC DC Protein Assay (Bio-Rad).

### Two-Dimension Gel Electrophoresis

Ten μg of protein extracts diluted in the IEF buffer (8 M urea, 2 M thiourea, 4% w/v CHAPS, 65 mM DTE, 0.0025% v/v bromophenol blue and 1% v/v IPG-buffer, pH 3-10 NL) were incubated for 1 h at room temperature on a rotating wheel. Precast IPG Strip (7 cm) with immobilized pH 4–7 gradient (Bio-Rad) were rehydrated with 125 μl of samples overnight. Isoelectric focusing (IEF) was carried out on an Ettan^TM^IPGphor^TM^ system (GE Healthcare) at 20°C using a gradient mode to a total amount of 8000 Vh. After IEF, proteins were reduced in equilibration buffer (65 mM DTT in 6 M urea, 1.5 M tris–HCl, pH 8.8, 30% v/v glycerol, 2% v/v SDS, and 0.001% v/v bromophenol blue) for 10 min, and then alkylated in equilibration buffer containing 135 mM iodoacetamide instead of DTT for 10 min. The proteins were separated in the second dimension on homemade 12% SDS-polyacrylamide gels using the Criterion Dodeca Cell system (Bio-Rad) at a constant voltage of 120 V during 80 min. Analytical gels were stained with silver nitrate. Three biological replicates per conditions and per strain were performed.

### Image Analysis and Spot Selection

Gels were scanned (Epson perfection V750 Pro) and images were analyzed with the Progenesis Samespot^®^ software v3.0 (Nonlinear Dynamics). After accurate gel alignment, the protein spots were detected automatically, and the quality of the automatic match was critically evaluated for each gel and, if necessary, corrections were made manually to eliminate any error or artifact in the protein spot assignment. Intensity values expressed as a percentage of normalized volumes are considered for further analysis.

### Spot Picking and Digestion in Gel With Trypsin

Each selected protein spot was excised from preparative gels and washed successively with water, 50 mM triethylammonium bicarbonate (TEABC) and 100% acetonitrile. Dried protein spots were rehydrated in 50 mM TEABC containing 0.1 μg of Sequencing grade modified trypsin (Promega) and incubated at 37°C overnight. Digested peptides of each gel piece were extracted successively with 100% acetonitrile, 50 mM TEABC and 5% formic acid and the supernatant were recovered into a new tube. The pooled peptide solutions were stored at −20°C until use.

### Mass Spectrometry and Protein Identification

The protein digests were analyzed using a Q Exactive Plus mass spectrometer (Thermo Fisher Scientific) interfaced with a nano-flow HPLC (RSLC U3000, Thermo Fisher Scientific). Samples were loaded onto a 15 cm reverse phase column (Acclaim Pepmap 100^®^ C18, Nano Viper, Thermo Fisher Scientific) and separated using a 30 min gradient of 2 to 40% gradient of buffer B (80% ACN, 0.1% formic acid) at a flow rate of 300 nl min^–1^. MS/MS analyses were performed in a data-dependent mode. Full scans (375–1,500 m/z) were acquired in the Orbitrap mass analyzer with a 70,000 resolution at 200 m/z. The twelve most intense ions (charge states ≥ 2) were sequentially isolated and fragmented by HCD (high-energy collisional dissociation) in the collision cell and detected at 17,500 resolution. The spectral data were analyzed via the Proteome Discoverer^TM^ v1.4.1.12 software (Thermo Fisher Scientific). For protein identifications, databases used were: *Exiguobacterium* sp. (strain ATCC BAA-1283) (UniProt Proteome ID: UP000000716_EXISA-cano_2017_11.fasta) with the subsequent parameters: Trypsin as the enzyme, one missed cleavage allowed, carbamidomethylation of cysteine as fixed modification and oxidation as variable modification. The MS identification of all proteins follows some filters: Mascot significance Threshold (Significance threshold: 0.01) and peptides per protein (minimal number of peptides: 1, count only rank 1 peptide: true, count peptide only in top scored proteins: true). All the proteomics data have been deposited to the ProteomeXchange Consortium (Deutsch et al., 2016) via the PRIDE partner repository (Perez-Riverol et al., 2018) with the dataset identifier PXD01470.

### Relative Gene Expression

To determine the relative expression of coding genes of identified proteins, we quantified transcripts levels by qRT-PCR. Bacterial strains were grown on the same conditions described previously and RNA extractions were carried out using the GeneJET RNA Purification Kit (Thermo Fisher Scientific) according to manufacturer’s instructions. RNA integrity, quality, and quantity were verified using 1% agarose electrophoresis, OD_260/280_ ratio and the QuantiFluor RNA System (Promega^®^). cDNA was synthesized using the M-MLV Reverse Transcriptase kit (Promega^®^) and random primer oligonucleotides hexamers (Invitrogen^TM^). The PCR reaction was carried out as follows: 10 min at 95°C followed by 40 amplification cycles (95°C × 30 s, 58°C × 30 s, and 72°C × 30 s), and a final step of 95°C × 15 s; 25°C × 1 s; 70°C × 15 s, and 95°C × 1 s) using specific primers for each gene (Supplementary Table S1). Transcript levels were quantified using the Brilliant II SYBR Green qPCR Master mix kit (Agilent Technologies) on a Stratagene Mx3000P thermal cycler. Gene expression levels were calculated according to Pfaffl (2001) using 16S rRNA gene as normalizator.

### *Exiguobacterium* Genomic Analysis

The arsenic resistance *ars* operon genetic context, including *cdr* gene was visualized using Genious^®^ v7.1.9 software. Using SH31 strain as reference (UniProt: UP000177837; NCBI: LYTG01000000) for BLASTP (Altschul et al., 1990); we queried the studied strains plus others from GenBank (Supplementary Table S2) to compare sequence identity of the genes related to *ars* operon and arsenic resistance (presence and copy number was evaluated with *E*-value 1E^–5^ and 80% identity).

### Western Blotting

Western Blot analyses were performed to validate expression changes of the identified LuxS protein detected on the three strains 2D-GE in response to both arsenic conditions. Samples were prepared as described previously and 50 μg of protein supernatant were loaded for SDS-PAGE, followed by its blotting onto a nitrocellulose membrane (Mini-PROTEAN Tetra Cell/Blotting Module – Bio-Rad). LuxS protein was probed primary with a rabbit polyclonal anti-LuxS antibody (1:5000 dilution; MyBioSource: MBS1491336) and a mouse anti-rabbit AP-conjugated was used as secondary antibody (1:5000 dilution; Abcam: 99696). Membranes were digitalized and analyzed with ImageJ v1.52a software (Schneider et al., 2012) using the digital densitometry values to calculate the expression as fold change.

### Statistics

All statistics and figures were computed using the “R/Bioconductor” statistical open source software (Gentleman et al., 2004), Progenesis Samespot^®^ software v3.0 (Nonlinear Dynamics) and GraphPad v5.0 (Prism^®^). The differential intensity levels of protein spots among the three conditions for each strain were analyzed using the R package Limma, an empirical Bayesian method for two group comparison similar to a two-sample *t*-test, except that a moderated t-statistic is calculated in which posterior residual SDs replace ordinary SDs (Ritchie et al., 2015). Differential relative gene expression for all comparison were analyzed using One-way ANOVA with post-hoc Tuckey HSD. A *p*-value less than 0.05 and fold change of more or less than 40% was considered statistically significant for all comparisons. Correlation analyses were performed using the R package Corrplot 0.84 (Wei and Simko, 2017) and plots were made with R package ggplot2 (Wickham, 2016). Heatmap and Hierarchical Ascendant Clustering (HAC) analysis was performed by using heatmap function of R package stats. HAC is a method of cluster analysis based on a pairwise distance matrix, which builds a hierarchy of clusters with sequentially agglomerative and divisive approaches. We used this method to organize the map and to group the statistically significant spots according to the nearest level of intensity. For this analysis, Euclidean distance and complete linkage were chosen as parameters.

## Results

### Bacterial Strains and Environmental Conditions

**FIGURE 1:**
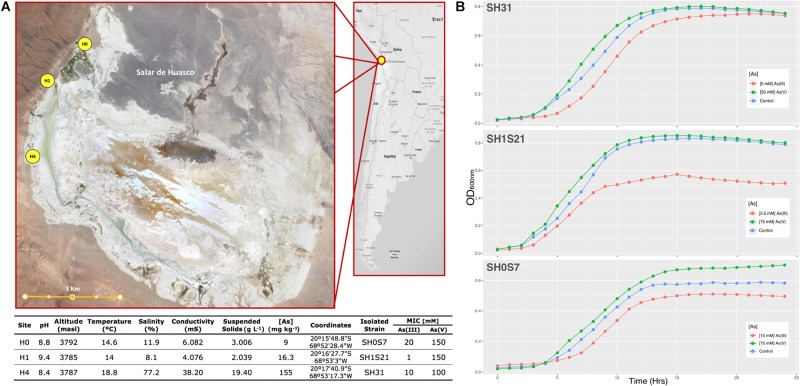
Sampling sites, strains characterization and As resistance. **(A)** Above: Salar de Huasco map showing the three sites locations (H0, H1, and H4), from which studied strains SH31, SH1S21, and SH0S7 were isolated (source: Google-Earth). Below: Parameters table of the three sites, sediment samples and isolated strains resistance levels. **(B)** Growth curves of the three studied strains, under the tested conditions: control, As(III) and As(V) at different concentration depending of the strains. OD_600_ readings were recorded during 24 h. Mean values (*n* = 3) are plotted.

Strains were isolated from different sites in the Salar de Huasco and those showed contrasting tolerance levels to As(III) and As(V). Each site had differential and specific environmental conditions (Figure 1A), it is mostly formed by shallow lagoons, permanent and non-permanent ponds and salt crusts (Uribe et al., 2015). The main differences among the three sampling points were salinity and arsenic concentrations. Salinity appears to increase gradually from north to south, as well as the As concentration with 9 mg kg^–1^ for H0 site, 16.3 mg kg^–1^ for H1 and 155 mg kg^–1^ for the H4 site, considering the distance between H0 and H1 is 1.6 km, between H1 and H4 is 2.4 km (the total distance from H0 to H4 is 4 km). Even though these samplings sites have been previously described (Dorador et al., 2008; Remonsellez et al., 2018), here we report for the first time the concentration and spatial variation of arsenic in the Salar de Huasco.

Analysis of the 16S rRNA gene sequence of the three strains revealed that SH0S7 has 98% identity with AC-CS-C2 strain (FJ231171) and SH1S21 strain has 97% identity with SH31 strain (LYTG00000000.1). The Average Nucleotide Identity (97.4% SH31 – SH1S21; 99% SH31 – SH0S7; 97.4% SH1S21 – SH0S7 and an average of 94.6% between S17 and our three strains) evidenced that our three strains are very close and related, they would belong to the same species as well as the S17 strain (Ordoñez et al., 2013). These results imply that their phenotypes are not related to species classification or taxonomy.

We monitored the capacity of these strains to grow under the experimental conditions [As(III) and As(V) at concentrations corresponding to half of the MIC] (Figure 1B) and we found that in the three strains, As(III) generated a greater effect on the kinetic and growth patterns of the strains (Supplementary Table S3). It is worth mentioning that, under all conditions, the bacteria were able to grow efficiently and the presence of every phase of bacterial growth is clearly observed. In the strains SH31 and SH1S21, it can be observed that cultures reached OD_600_ of 0.4 in around 6 h. However, SH0S7 strains needed around 10 h to reach the same OD. This is probably due to a different requirements or ability to adapt to the culture conditions independently of the As concentrations.

### Comparison of *Exiguobacterium* Proteomic Profiles in Response to Arsenic Species

**FIGURE 2:**
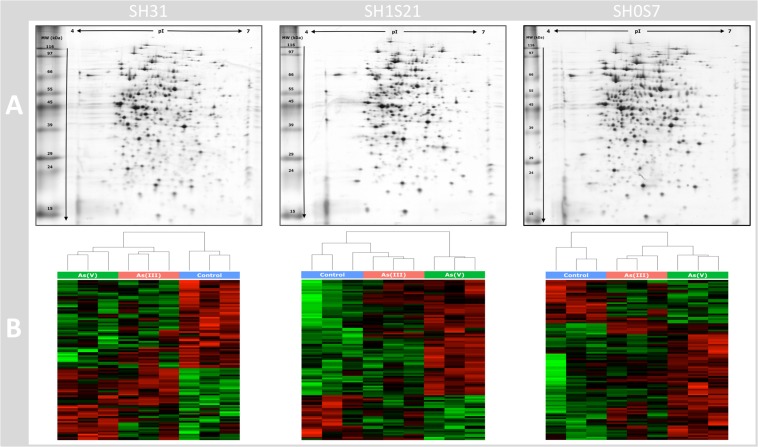
Proteomic analysis of the three studied strains. **(A)** 2D-GE of proteins extracted from supernatant of bacteria culture in As(V) condition for each *Exigobacterium* strain showed as example. **(B)** Heat maps of the top 100 protein spots by expression levels, present in all 2D-GE samples (three strains in three conditions in triplicate). The intensity levels (percentage of normalized volumes) of the protein spots were visualized by a heat map, according to their statistical significance *p* of the Limma analysis, which were plotted using ggplot2. Each column represents the data from one 2D-GE experiment. Rows represent individual spots and graduated scale color codes from green (low level of intensity) to red (high level of intensity).

To identify proteins that could be involved in the tolerance of bacteria to the toxicity and stress generated by the presence of arsenic, we analyzed by 2D-GE the proteomic profiles of each strain in the three conditions: control, As(III) and As(V) (examples of 2D-GE gels were showed in Figure 2). After image analysis, 999 protein spots were detected from proteins extracts with a wide range of molecular weight (20–200 kDa) and isoelectric points (4–7 pI). Various comparisons were performed to select informative differences in protein expression: comparison of control conditions, As(III) and As(V) for each strain, comparisons of control conditions separately, As(III) and As(V) for all strains.

The comparison to the control condition to As(III) and As(V) conditions allowed to identify, respectively, 173 and 90 differential protein spots expressed in SH31 strain, 111 and 143 in SH1S21 strain and 77 and 120 for SH0S7. We observed more differential protein spots expressed between control vs. As(V) for the SH1S21 and SH0S7 strains than control vs. As(III) comparison. This was the opposite for the SH31 strain. It is not clear what are the reasons for these differences between the three strains response patterns.

In order to better visualize the protein expressions, the “100 best” protein spots, statistically different in each strain, were subjected to hierarchical clustering analysis generating the heat map shown in Figure 2. We observed that replicates are correctly grouped by conditions in each strain. Data distribution according to selection criteria is shown in the Supplementary Figure S1. Then we selected the most relevant protein spots (statistically and manually verified), from all comparisons for mass spectrophotometry identification. Several stringent criteria to select protein spots for MS identification were imposed: statistical significance (*p* ≤ 0.05) and fold change (FC ± 40%), differential protein spot expression should be change and in the same way in the triplicate samples and inter-strains comparison were also taken in consideration, meaning that we took into account, whether if the spot was present in one strain (regardless significative expression change) but absent in another. All selected spots were manually validated.

### Protein Identification

A total of 27 protein spots were selected and 25 different proteins were identified by MS (Table 1). Most of the identified proteins were assigned to four categories according to its function in the cell: metabolism (GcvPB, PdhA, GlpX(2), SucD, ArcC, PfkA, TpiA, and DhaL), synthesis [CarB, GlnE, WecC, PdxS(2), PunA, and GlmS], stress response (DnaK, Cdr, A0A1G1SP02, Hpf, LuxS, and Fur), transport (ArsA, A0A1G1SJA6, YxdL, and ArtM) and one hypothetical protein.

**TABLE 1:** Identification of the protein spots selected in response to As.

**Spot**	**UniProt^a^**	**Protein**	**Experimental pI/**	**Mascot**	**GenBank**	**SH31 genome^b^ function**
	**accession**		**MW (kDa)**	**score**	**accession**	
**Metabolism**						

893	A0A1G1SNT0	Probable glycine dehydrogenase	5.62/53.90	30.39	WP_071397724.1	GcvPB: Glycine dehydrogenase
1255	A0A1G1SHJ5	Pyruvate dehydrogenase E1 component	5.06/40.50	181.22	WP_070327597.1	PdhA: Pyruvate dehydrogenase E1
1282	A0A1G1SN38	Fructose-1,6-bisphosphatase	4.88/34.90	213.54	WP_071397901.1	GlpX: Fructose-1,6-bisphosphatase
1308	A0A1G1SH54	Succinate–CoA ligase	5.14/31.60	28.95	WP_070327506.1	SucD: Succinate–CoA ligase
1355	A0A1G1SLJ5	Fructose-1,6-bisphosphatase^∗^	5.10/34.40	445.20	WP_071398377.1	GlpX: Fructose-1,6-bisphosphatase^∗^
1356	A0A1G1SL84	Carbamate kinase	5.19/33.90	111.47	WP_071398490.1	ArcC: Carbamate kinase 2
1382	A0A1G1SNF1	ATP-dependent 6-phosphofructokinase	5.20/34.30	352.42	WP_084812917.1	PfkA: ATP-dependent 6-phosphofructokinase
1695	A0A1G1SN06	Triosephosphate isomerase	4.81/26.80	933.53	WP_070328703.1	TpiA: Triosephosphate isomerase
1852	A0A1G1SN98	Dihydroxyacetone kinase	4.83/21.20	81.73	WP_071397851.1	DhaL: PTS-dependent dihydroxyacetone kinase

**Synthesis**						

369	A0A1G1SHA6	Carbamoyl-phosphate synthase	4.98/117.60	49.13	WP_071399568.1	CarB: Carbamoyl-phosphate synthase
1001	A0A1G1SP81	Glutamine synthetase	5.06/50.00	2377.84	WP_071397634.1	GlnE: Glutamine synthetase
1080	A0A1G1SMV3	UDP-N-acetyl-D-mannosamine dehydrogenase	5.12/46.90	677.40	WP_071398119.1	WecC: UDP-N-acetyl-D-mannosamine dehydrogenase
1466	A0A1G1SKT1	Pyridoxal 5′-phosphate synthase	5.60/32.00	2367.00	WP_070329506.1	PdxS: Pyridoxal phosphate synthase
1480	A0A1G1SKT1	Pyridoxal 5′-phosphate synthase^∗^	5.60/32.00	64.18	WP_070329506.1	PdxS: Pyridoxal phosphate synthase^∗^
1531	A0A1G1SP27	Purine nucleoside phosphorylase	4.92/29.30	133.59	WP_016509380.1	PunA: Purine nucleoside phosphorylase
2310	A0A1G1SL85	Glutamine–fructose-6-phosphate	5.15/65.20	261.25	WP_071398579.1	GlmS: Glutamine–fructose-6-phosphate aminotransferase

**Stress response**						

749	A0A1G1SNI7	Chaperone protein DnaK	4.86/65.30	1313.23	WP_071397788.1	DnaK: Chaperone protein
831	A0A1G1SMS6	Dehydrogenase	5.41/59.70	433.32	WP_071398091.1	Cdr: Coenzyme A disulfide reductase
1571	A0A1G1SP02	Metallophosphoesterase	5.85/29.40	32.49	WP_071397651.1	YmdB: 2′,3′-cyclic-nucleotide 2′-phosphodiesterase
1894	A0A1G1SMF5	Ribosome hibernation promoting factor	5.39/21.60	47.06	WP_016508657.1	HpF: Ribosome hibernation promoting factor
2158	A0A1G1SNP5	S-ribosylhomocysteine lyase	5.21/17.70	67.48	WP_016508081.1	LuxS: S-ribosylhomocysteine lyase
2308	A0A1G1SP20	Transcriptional repressor	6.33/19.90	56.59	WP_016509373.1	Fur: Ferric uptake regulation protein

**Transport**						

664	A0A1G1SMT2	Arsenical pump-driving ATPase	5.06/64.80	759.07	WP_071398094.1	ArsA: Arsenical pump-driving ATPase
1543	A0A1G1SJA6	Bacitracin ABC transporter ATP-binding protein	6.55 / 31.50	49.73	WP_071399005.1	Fluoroquinolones export ATP-binding protein
1677	A0A1G1SM10	Bacitracin ABC transporter ATP-binding protein	5.03/27.70	103.06	WP_071398199.1	YxdL: ABC transporter ATP-binding protein
1751	A0A1G1SNV0	Peptide ABC transporter ATP-binding protein	5.52/26.40	28.44	WP_071397699.1	ArtM: Arginine transport ATP-binding protein

**Others**						

525	A0A1G1SM64	Uncharacterized protein	5.19/101.60	29.85	WP_071398150.1	Hypothetical protein

*All protein spots were identified by *Exiguobacterium* sp. ATb1 protein database. a: Identified proteins in *Exiguobacterium* sp. SH31 Uniprot Proteome. b: Identified protein sequences were queried through BlastP against *Exiguobacterium* sp. SH31 Prokka Annotated proteins (.faa: protein multifasta file). ^∗^: two different spots were identified as the same protein.*

**FIGURE 3:**
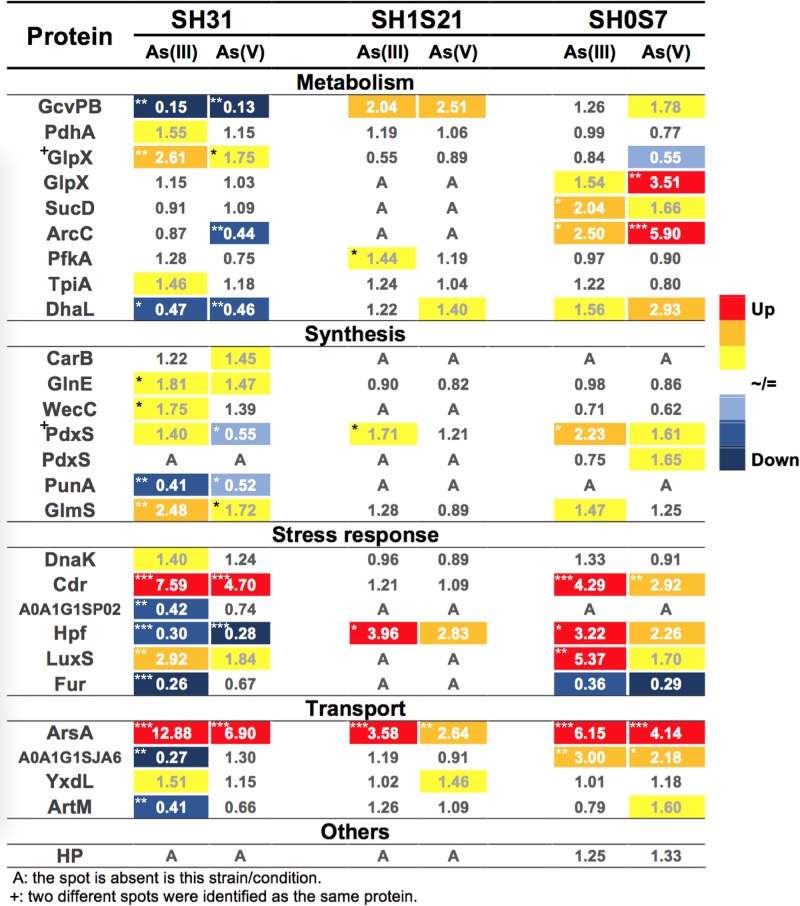
Expression heat map of the identified proteins. Table shows protein expression levels in response to arsenic conditions for the three studied strains. Number indicates fold change for As(III) and As(V) regarding control condition. Each column represents the mean data of three replicate for each As condition. The graduated scale color codes were from blue (low level of intensity) to red (high level of intensity), asterisks represent statistical significance. ^∗^*p* ≤ 0.05; ^∗∗^*p* ≤ 0.01; and ^∗∗∗^*p* ≤ 0.001.

The regulation of proteins involved in the response to arsenic treatment is different depending on the studied strain as shown in Figure 3. For instance, in *Exiguobacterium* sp. SH31 the robust up regulation occurs on ArsA and Cdr proteins in both As conditions. The detection of ArsA protein in response to arsenic is described here for the first time for the *Exiguobacterium* genus. On the other hand, the up regulation of Cdr protein would demonstrate the activation of the antioxidant mechanism, necessary for counteracting the oxidative stress caused by arsenic (Weiss et al., 2009; Ciprandi et al., 2012; Slyemi and Bonnefoy, 2012; Kruger et al., 2013).

The response to As in this strain is also manifested on several proteins related to metabolism (Figure 3). For instance, the GlpX protein is a fructose-1,6-bisphosphatase and was up-regulated by both species of As. This enzyme would generate Pi to save energy, in response to phosphate transport regulation (to avoid As influx) (Movahedzadeh et al., 2004; Elias et al., 2012), and generate reducing power in the form of NADPH throught the pentose phosphate pathway (Nelson and Cox, 2008). Additionally, the glutamine–fructose-6-phosphate aminotransferase (GlmS) was up-regulated by both As species, this protein catalyzes the first step in the hexoamines pathway using fructose-6P to produce glucosamine-6P, which is a precursor for cell wall synthesis. Another protein that was significantly up-regulated in response to As(III) is WecC (UDP-N-acetyl-D-mannosamine dehydrogenase). WecC participates in the synthesis of polysaccharides that could be involved in the biofilm production, a known defense strategy. The extracellular matrix has also been reported as a strategy for sequestering of toxic compounds, as they get trapped in the structure (Weiss et al., 2009).

The pyruvate dehydrogenase E1 component (PdhA) was up-regulated only in response to As(III), this was previously described in *Staphylococcus* exposed to As(V) (Srivastava et al., 2012). The ATP-dependent 6-phosphofructokinase (PfkA) that catalyzes the fructose-1,6-bisphosphate formation is down-regulated only by As(V), corresponding to the cell stress state and the need for reductant generation and energy salvage. This is consistent with the slight increase in the triosephosphate isomerase (TpiA) in As(III), that generates glyceraldehyde 3-phosphate, indicating activation of late stage of glycolysis, with reductants gaining (Trujillo et al., 2014). The dihydroxyacetone kinase subunit L (DhaL) and the glycine dehydrogenase subunit 2 (GcvPB) were found to be significantly down-regulated in both arsenic conditions. The carbamate kinase (ArcC) was down-regulated in both arsenic condition but it is only significant in As(V) condition. The glutamine synthetase (GlnE) is a negative regulator of glutamine synthesis concerning to cell nitrogen levels and it was up-regulated in both As conditions (Leigh and Dodsworth, 2007), suggesting an increase in energy generation. As a whole these changes in expression level of different metabolic pathways demonstrate that energy generation is one of the bacterial strategies to overcome As related stress (Nyström, 2004).

Pyridoxal phosphate has been previously associated with the response to several stress conditions mainly acid, osmotic and as an important factor against oxidative stress (Han et al., 2005; Schnell et al., 2015; Xie et al., 2017). Here we found, that a subunit of the pyridoxal 5′-phosphate synthase (PdxS) was significantly down-regulated in response to As(V) and up-regulated in presence of As(III). This compound is a co-factor in the cysteine synthesis which is a key component of thiols (antioxidants) (Nelson and Cox, 2008; Schnell et al., 2015). Also, purine nucleoside phosphorylase (PunA) was down-regulated in As(III) and As(V) condition, this enzyme participates in the formation of nucleic acid precursors and has also been demonstrated that during stress they can become sources of energy, carbon and nitrogen (Kilstrup et al., 2005).

Among the proteins identified as related to stress response, the S-ribosylhomocysteine lyase (LuxS) was found to be over expressed in both As conditions, LuxS function is associated with quorum sensing and biofilm formation. The transcriptional repressor (Fur), ribosome hibernation promoting factor (Hpf) and a metallophosphoesterase (A0A1G1SP02) are down-regulated in both As conditions as previously reported (Pandey et al., 2012; Belfiore et al., 2013; Sacheti et al., 2014). DnaK chaperon was only up-regulated in this strain on As(III) condition. In previous works, DnaK protein was detected as part of arsenic response as well as other stress situations, participating in misfolded and aggregated proteins recovery (Zhang et al., 2007; Daware et al., 2012; Belfiore et al., 2013).

In regards to transport, the YxdL protein, associated to the bacitracin resistance was increased in response to As(III). Transport through the membrane is one of the most common mechanisms to avoid toxicity used by bacteria (Nies and Silver, 1995). The proteins of cellular transport were strongly down regulated in *K. pneumoniae* in response to As(III), to avoid toxic influx (Daware et al., 2012). On the contrary, the transporters ArtM and A0A1G1SJA6 were down-regulated, this could be a way of preventing toxic influx as aforementioned.

Another strain studied was *Exiguobacterium* sp. SH1S21, that showed contrasting results compared to SH31, namely, the absence of protein spots corresponding to SucD, ArcC, CarB, WecC, PunA, LuxS, Fur, A0A1G1SP02 and to GlpX and PdxS protein spots (only on one of the two protein spots) on both As conditions, evidencing clearly a different pattern. On the other hand, the strong induction of GcvPB, Hpf, and ArsA in both As conditions was observed. The identified proteins were either absent or up-regulated but no significantly down-regulated in SH1S21. ArsA demonstrates an active response against arsenic as aforementioned. The expression of the enzymes that participate on transcription and translation such as Hpf has been proposed as cellular stress indicators, because protein biosynthesis is increased in response to adverse conditions (Sacheti et al., 2014). Furthermore, the glycine cleavage system (GcvPB), in addition to generate helpful molecules from glycine could also generate important reductants against As associated oxidative stress (Okamura-Ikeda et al., 1993). Moreover, PfkA and PdxS were up-regulated in As(III) while YdxL and DhaL were only over expressed in As(V). DhaL catalyzes the formation of dihydroxyacetone phosphate and pyruvate that would be feeding into the carbohydrates metabolism for energy generation (Gutknecht et al., 2001). It is worth mentioning that the regulation of PfkA and YdxL proteins is contrary to that observed in SH31 strain, suggesting the relevance of a different defense mechanism in this strain.

Finally, comparing *Exiguobacterium* sp. SH0S7 with the SH31 strain, the most relevant findings are: the detection of a second PdxS protein exclusively on this strain, being repressed in As(III) but induced in As(V); the presence of an hypothetical protein, although has no significant change is exclusive for this strain, which is the one with the highest As tolerance among the three. The PdxS protein identified only in SH0S7 is regulated opposite to the PdxS protein identified in SH31 according to As conditions. This observation suggests a modification of the PdxS protein that is dependent on the arsenic condition and the strain, however, this remains to be demonstrated. Moreover, another exclusive finding for this strain is the induction of the A0A1G1SJA6 transporter in both conditions, which is poorly characterized and could be a determining factor on the great tolerance of this strain, this protein is a good candidate for further investigation. The induction of ArsA, Cdr, LuxS, Hpf and the repression of Fur supports our previous statements about an active response against arsenic and its associated stress.

Additionally, other up-regulated proteins, in both As conditions and involved in metabolism cell were GlpX, DhaL, ArcC and SucD. The carbamate kinase (ArcC), is associated with several pathways, such as the metabolism of purines, proline, arginine, and glutamate (important part of glutathione), carbamoyl phosphate can be used as a source of phosphate in glycolysis and the energy metabolism, it has also been reported as part of osmotic stress response in several bacteria (Casiano-Colón and Marquis, 1988; Ceylan et al., 2012). On the other hand, Carbamoil-fosfato sintasa I (CarB) is absent in this strain regarding SH31, and it produces carbomyl phosphate with a greater energy cost which would be inconvenient for the cell under As stress (Nyunoya and Lusty, 1983). Also, Succinate–CoA ligase (SucD) participates in the TCA cycle, in a step that produces energy in form of ATP/GTP and CoA-SH as reducing agent (Joyce et al., 1999). Proteins related to energy metabolism were the most enriched from the ones that were differentially expressed. Comprising proteins that participate in pathways like pentose phosphate, glycolysis, amino acid metabolism that would be in accordance to the necessary requirements for the bacteria to prosper under As-generated stress.

**FIGURE 4:**
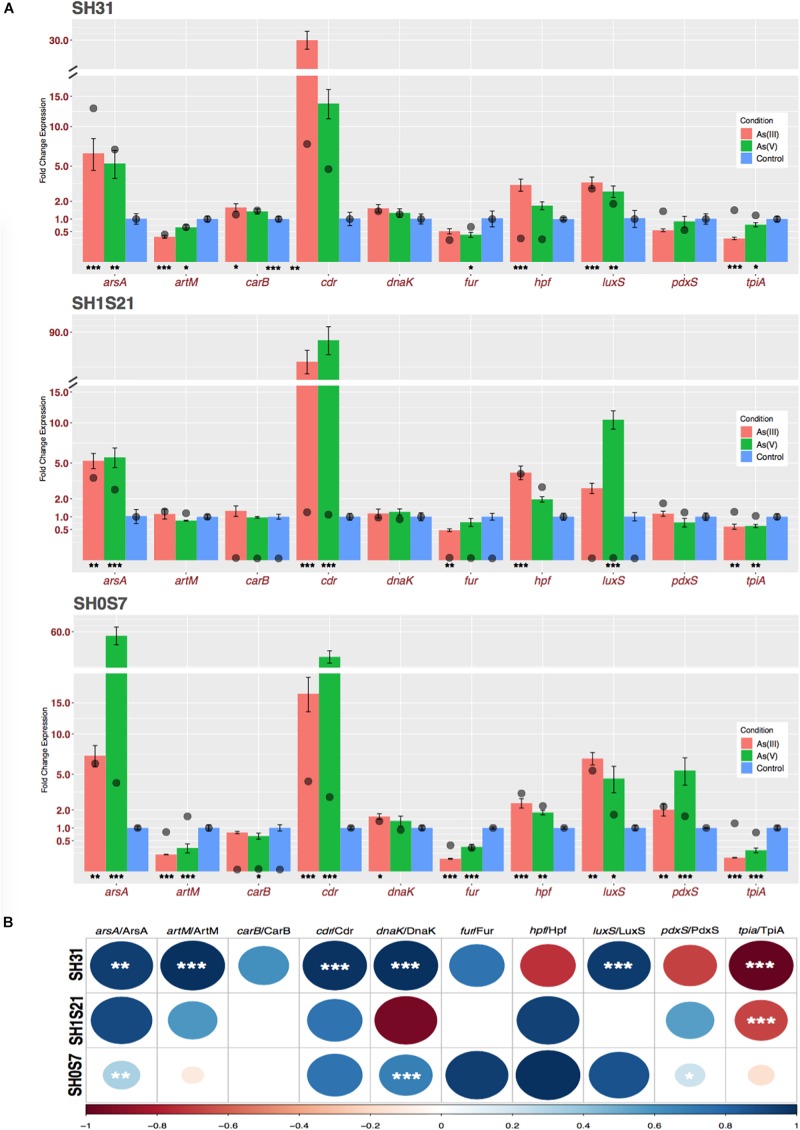
Transcriptomic expression of genes that code for the identified proteins. **(A)** Gene and protein fold change expression: Bars represents relative fold change gene expression related to control condition of selected genes, measured under the same As conditions as in the proteomic experiments (colored by condition). Plotted data is an average of three independent experiments with three technical replicates each. Asterisks represent statistical significance of transcriptional expression experiments both As compared to control condition. The protein mean fold changes (three independent experiments) obtained from proteomic analysis are represented by the black circles. The data for both As conditions are represented relative to the control values. **(B)** Correlation index between transcriptional and protein fold change expression values. Positive correlations are displayed in blue and negative correlations in red color. Color intensity and circle size are proportional to the correlation coefficients; asterisks represent statistical significance. ^∗^*p* ≤ 0.05; ^∗∗^*p* ≤ 0.01; and ^∗∗∗^*p* ≤ 0.001.

Finally, in order to validate the protein regulation observed we have studied the regulation level of protein transcripts. For that, we carried out qRT-PCR quantification of the transcripts for ten of the identified proteins (Figure 4A). In the comparison of As(III) and As(V) conditions to control condition, we observed a statistically significant regulation of 8, 6, and 10 transcripts studied for the SH31, SH1S21, and SH0S7 strains, respectively. The protein and transcript expression levels were compared, and a correlation index was calculated. The mRNA levels are overall well correlated at the protein level, the TpiA transcript/protein which has the most notorious negative correlation (Figure 4B).

### Signaling Pathway and Process Analyses of Identified Proteins

The proteins identified in the supernatant of *Exiguobacterium* bacteria subject to arsenic conditions might reflect altered biological processes and pathways. To carry out functional analysis, the 25 differentially expressed proteins were subjected to gene ontology and protein-protein interaction analysis. From these protein sets, a total of five biological processes were enriched in Gene Ontology analyzed by Ontologizer (Bauer et al., 2008) and PANTHER (Mi and Thomas, 2009). The most obvious enriched categories (*p*-value and ratio observed genes/total genes described in the process) were fructose 6-phosphate metabolic process (GO:0006002) and aldehyde biosynthetic process (GO:0046184). The proteins assigned to these two categories were PfkA (both), GlmS and TpiA, PdxS, respectively. The fructose 1,6-bisphosphate metabolic process (GO:0030388) was also identified as co-occurring terms of fructose 6-phosphate metabolic process (GO:0006002) with two proteins assigned: GlpX, PfkA. The glycolysis (P00024) was the only pathway enriched in PANTHER. For the protein-protein interaction analysis, we built a wider network by looking at connected proteins (first-degree neighbors) with our set of 25 identified proteins using the merge network imported from Cytoscape v3.6.0 (Shannon et al., 2003). Only one highly connected “hub” protein was identified: catabolite control protein A (CcpA) connecting six identified proteins (Supplementary Figure S2).

### Genomic Context of *cdr*

**FIGURE 5:**
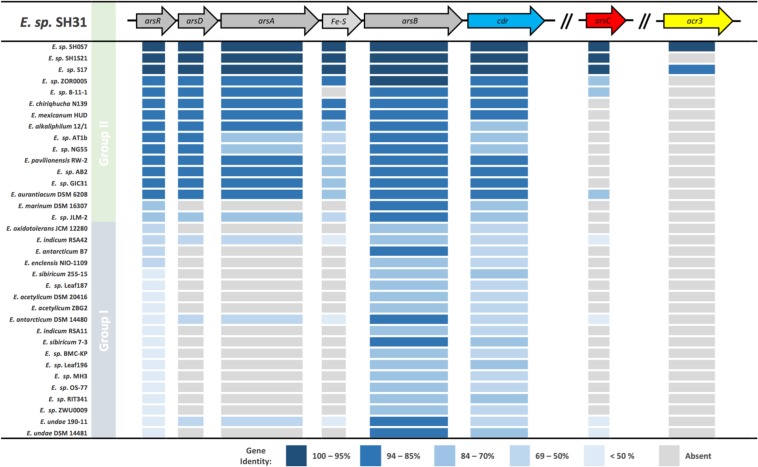
Genetic organization of relevant arsenic resistance genes. Genes of interest in *Exiguobacterium* strains: *arsR* arsenical resistance operon repressor; *arsD* arsenical resistance operon *trans-*acting repressor; *arsA* arsenical pump-driving ATPase; Fe–S putative iron sulfur protein; *arsB* arsenical pump membrane protein; *cdr* coenzyme-A disulfide reductase; *arsC* arsenate reductase; *acr3* arsenite efflux pump. Heat scale shows identity percentage of all strains sequence compared, using as reference the *Exiguobacterium* sp. SH31 genes. Grouped according to Vishnivetskaya et al. (2009).

The *cdr* gene encodes for a coenzyme A disulfide reductase (dehydrogenase), which has been associated with oxidative stress response. Interestingly, exploring the SH31 strain genome we found that the *cdr* gene is located within the genomic context of the *ars* operon. The same contexts were observed in the SH1S21 and SH0S7 strains genomes (GenBank accessions: SH1S21: GCA_004337175.1 and SH0S7: GCA_004337195.1). This shows a possible association of its function with the As(V) detoxification mechanism. This relationship has never been described before. In order to verify if this statement is valid for the genus, we carried out a comparative analysis of the *ars* operon (and arsenic related genes *arsC* and *acr3*) of these three strains in addition to 33 other *Exiguobacterium* genomes available in GenBank (Supplementary Table S2). This result led us to conclude that independent of the operon composition (*arsRDAB* or *arsRB*) and the differential tolerance level to As, the gene *cdr* is present on every case downstream from the gene *arsB* (Figure 5). It is interesting to observe that the *arsC* reductase seems to be more associated to the strains of group 2 and the *acr3* accessory pump would be more related to the altiplanic strains or those with a higher level of As resistance, as reported before (Castro-Severyn et al., 2017). Strikingly, the genetic sequence of the *acr3* accessory pump is not present in SH1S21 strain, which could be corresponding to the low As resistance level presented by this strain compared to the other two.

### Quantification of the LuxS Protein

To validate the LuxS protein regulation observed in 2D-GE, we carried out the quantification of the LuxS at protein level through western blot as well as it was previously validated at transcript level. An up regulation of LuxS was observed for As(III) condition compared to the control conditions (1.47 and 1.38, for SH31 and SH0S7, respectively). This regulation is weaker, in As(V) condition (1.21 and 1.27 for SH31 and SH0S7, respectively). These results confirm the proteomic data of SH31 and SH0S7 strains with an upregulation in both As conditions (Supplementary Figure S3). In SH1S21 strain, LuxS expression level did not vary in any arsenic condition compared to the control [0.76 and 0.91, As(III) and As(V), respectively], being non-correlative neither with proteomic observation nor with the transcriptional gene expression. These results support our findings and could imply relevance of LuxS protein in the capacity of the studied strains to tolerate arsenic.

## Discussion

The organisms living in the different environments found in high-altitude Andean lakes have to tolerate/resist diverse extreme conditions as well as the co-occurrence of these (Albarracín et al., 2015, 2016). Among the most relevant conditions for this area are desiccation, temperature variation, osmotic pressure, solar radiation and mineral composition which is very variable, including, in most cases, toxic components such as arsenic (Risacher and Fritz, 2009; Uribe et al., 2015; Hernández et al., 2016). Specifically, the three sampled spots in the Salar de Huasco showed variability in regards to physicochemical parameters (Figure 1A). Salinity is one of the most influencing factors that pressure and shape microbial communities’ composition (Canfora et al., 2014; Kimbrel et al., 2018). In Salar de Huasco the great microbial diversity and its variation within the area have been previously described (Dorador et al., 2008, 2010). In this relatively small area, salinity variation goes from freshwater to hypersaline water, which would contribute to diversity. This is in accord to the salinity values that we found, from 8.1% in H1 to 77.2% in H4 collection spots (Figure 1A).

Arsenic presence specifically in northern Chile is well documented, due to mining exploitation (mineral richness) and the great health problems associated (Ferreccio and Sancha, 2006; Demergasso et al., 2007; Roh et al., 2018; Tapia et al., 2018). In spite of this, only limited As measurements have been reported for Salar de Huasco (De Gregori et al., 2003; Herrera et al., 2009). Our results show an important variation of As concentration in the analyzed sediments, which increases proportionally from north to south, corresponding to the water flux model described by Hernández et al. (2016). Underground sources and superficial flow feed these water bodies, and aquifers that provide the underground water are refilled by seasonal rain percolation. Furthermore, mineral composition levels are similar between the streams and their underground sources. However, evaporation and natural flux cause a gradual accumulation in sediments by stratification and precipitation. This phenomenon explains the As increase on the southern site (H4), where the flow or shallow current is gradually weaker in comparison to the northern ones (Acosta and Custodio, 2008; Hernández et al., 2016).

Enrichment of bacteria capable of metabolizing arsenic compounds has been reported in Salar de Ascotan and Salar de Atacama, located also in northern Chile, where conditions are similar to those found in Salar de Huasco. This is related to the high salinity and As concentrations present in these environments, confirming the fact that a metabolism based on bio-energetically favorable electron acceptors such as As(V) becomes more important when the salinity is near the saturation level (Lara et al., 2012). Also, it has been reported the presence and enrichment of bacteria capable to precipitate As (as realgar and orpiment minerals) in brine and boron deposits (Demergasso et al., 2007).

The As(V) and As(III) tolerance levels shown for our three strains contrast with those previously reported for this genus: strains WK6 (Anderson and Cook, 2004), KM05 (Sunita et al., 2012), S17 (Belfiore et al., 2013), PS NCIM 5463 (Sacheti et al., 2014), AS-9 (Pandey and Bhatt, 2015), *antarcticum* B7 (da Costa et al., 2018), and *profundum* PT2 (Andreasen et al., 2018). We highlight the ability of SH0S7 strain, isolated from H0 site, to tolerate 20 mM of As(III), one of the highest concentrations reported so far.

The capacity to grow and tolerate the presence of high concentrations of As in all three strains of *Exiguobacterium* was evaluated using half of the determined MIC value for both As(V) and As(III) (Figure 1B). All strains grew on the presence of both As species and we calculated the specific growth rates (μ) (Blanch and Clark, 1997) and the generation time (GT) (Álvarez-Ordóñez et al., 2010) for each strain. We observed that the As(III) causes a greater effect as a delay growth in the three studied strains affecting significantly μ (diminished) and GT (increased) parameters (Supplementary Table S3). This was expected because it was previously reported the negative effect of As(III) on bacterial growth due to its higher toxicity (Petänen et al., 2003; Poirel et al., 2013). In regard to As(V), these parameters were less affected and even a slightly better growth rate was observed in SH1S21 and SH0S7 strains regarding control condition. This phenomenon is related to the fact that the bacteria can use the energy generated by As(V) reduction (Ordoñez et al., 2015).

Qualitative and quantitative differences in proteomic profiles were observed not only between control and As conditions for each strain, but also among the strains. Indeed, a global coherence between the strains proteomic patterns was not observed, considering the presence/absence of the identified spots and their expression levels (Figure 3 and Table 1). It is interesting to note that the levels of regulation reported in our study are much greater, compared to previously reported results, comprising a wide range of fold change values from 0.26 to 12.8 (Ciprandi et al., 2012; Daware et al., 2012; Belfiore et al., 2013).

The protein levels of a cell reflect the state of physiological response to the conditions in which it is found on a determined point in time, against any alteration (Cleiss-Arnold et al., 2010). Consequently, it is relevant to study the changes in protein patterns as effect of external pressures and understanding the state of the cell as a system. All differences observed between the three strains could be the result of their adaptations to their environment specific conditions and the identified proteins could have a relevant role in the arsenic tolerance level.

Each studied strain presents a slightly different protein expression pattern in response to As. In the *Exiguobacterium* sp. SH31 strain we found that the ArsA and Cdr proteins are expressed in both As conditions, this would suggest an active response to address arsenic toxicity (Slyemi and Bonnefoy, 2012), as this protein is part of *ars* operon and its expression is correlated to the activation of the whole system (Andres and Bertin, 2016). Additionally, Cdr has an important antioxidant role in organisms that do not have classic detoxification systems (Cárdenas et al., 2012; Mateos et al., 2017). Moreover, it has been found that CoASH is the major low-molecular-weight thiol in *Borrelia burgdorferi* being able to reduce H_2_O_2_ and then be regenerated by Cdr, supporting a role associated with ROS defense (Boylan et al., 2006). In regard to metabolism, key proteins such as GlpX and GlmS were up-regulated by both species of As, suggesting the requirement for Pi generation and avoid As influx; also, production of NADPH as a reductant molecule as well as cell wall synthesis. Additionally, polysaccharide synthesis (WecC) is affected by the presence of As(III), that could be related to biofilm production.

Also, PdhA was up-regulated only in response to As(III), suggesting part of the response to As is to generate reductants that could be counteracting As-associated oxidative stress (Nelson and Cox, 2008). In line with this results, the expression of PdxS is up-regulated in presence of As(III). This compound is a co-factor in the cysteine synthesis which is a key component of thiols (antioxidants) (Nelson and Cox, 2008; Schnell et al., 2015). Also, an increase in cell nitrogen levels associated with the upregulation of the GlnE is an indicator of an increase in energy generation, parts of these pathways can feed TCA cycle as it has been demonstrated in *Ferroplasma* in response to As(III) (Baker-Austin et al., 2007). The metabolic response to As has not been completely clarified given the fact that there are not enough data to support robust arguments and the investigations available have been done with very diverse organism from particular niches (Andres and Bertin, 2016). All these changes in expression of proteins related to metabolic pathways demonstrate that energy generation is one of the bacterial strategies to overcome As-related stress (Nyström, 2004).

As it was previously reported for *H. arsenicoxydans* and *Exiguobacterium* sp. S17 (Cleiss-Arnold et al., 2010; Belfiore et al., 2013) we found that PunA expression is repressed in As(III) and As(V) conditions. Also, LuxS, a general stress protein, associated with the control of controlling quorum sensing and biofilm formation (Sewald et al., 2007; Hardie and Heurlier, 2008; Remonsellez et al., 2018) is over expressed in both As condition. The resistance to As is increased by the activation of the global stress response, as well as the protection against metals/metalloid associated oxidative stress (Daware et al., 2012). The induction of proteins related to the iron uptake under As exposure has been previously reported (Zhang et al., 2016). Fe is an important cofactor that participated in several central metabolism, biosynthetic pathways and gene regulation (Hassan and Troxell, 2013). This phenomenon would correspond to the Fur protein down regulation in our study. In SH31, transport-related proteins, like YxdL was increased in response to As(III). Transport through the membrane is one of the most common mechanisms to avoid toxicity used by bacteria (Nies and Silver, 1995). The proteins of cellular transport were strongly repressed in *K. pneumoniae* in response to As(III), to avoid toxic influx (Daware et al., 2012). These changes in transport processes modify the pathways of anabolism and catabolism to compensate for the lack of biomolecules that in regular conditions are taken from extracellular media (Andres and Bertin, 2016). It has been widely reported that molecular transport is essential for bacteria survival during As-associated stress, for both intake and extrusion. However, detecting transporters in proteomics is challenging because they are probably insoluble and could be constitutively expressed, as reported by Belfiore et al. (2013) for ArsB and ACR3. Our results show that the genes for both transporters are in SH31 genome (Figure 5; Castro-Severyn et al., 2017) and here we demonstrated that the expression level of ArsA is increased at transcript and protein levels in response to As (Figure 4).

*Exiguobacterium* sp. SH1S21 shows a different expression pattern in some proteins, the activation of As response is evidence by the upregulation of ArsA, also, stress response proteins such as Hpf and GcvPB are also found to be upregulated. Additionally, we showed that the sequence of the *acr3* accessory pump is absent in SH1S21 strain and also Cdr, LuxS, Fur and ArcC proteins were not detected or not regulated in SH1S21 compared to SH31 and SH0S27 strains independently of As conditions. These results suggest a role of these proteins in the mechanisms involved in the low As resistance level presented by this strain.

Furthermore, *Exiguobacterium* sp. SH0S7, that has the highest As tolerance from the three strains, has a second PdxS protein detected exclusively on this strain, interestingly we found an induction of the A0A1G1SJA6 transporter in both conditions, this could be relevant to the ability to tolerate high concentration of the metalloid, and needs further investigation. Also, the induction of ArsA, Cdr, LuxS, Hpf and the repression of Fur is consistent with observations made in the other strains. Another important protein induced is the Carbamate kinase (ArcC) associated to key pathways. In this strain is also evidenced the importance of metabolic modulation under this stress, as during glycolysis, it has been found that As(V) is able to interfere with this metabolic process by the substituting PO_4_^–2^ inside the cell (Goyer and Clarkson, 2001; Andres and Bertin, 2016). The maintenance of the energy supply or the ability to adapt to other carbon sources is essential to ensure survival of the cell. In the same context, the protein CcpA which ensures optimal energy usage under diverse conditions (Tomoyasu et al., 2010) was identified as hub protein connecting six proteins, suggesting a rapid adaptation to energy utilization and prioritization of available carbon sources in response to arsenic. It has been previously found that oxidative stresses caused by arsenic resulted in altered levels of enzymes related to carbohydrate metabolism (Shahid et al., 2014).

Overall, one of the most prominent results obtained from the proteomic analysis was the detection of the arsenic pump-driving ATPase ArsA. Being up-regulated in both As conditions for the three strains, with a great magnitude against As(III) (Figure 3). In this case, transcriptional expression of the gene that codes for this protein showed a positive correlation. ArsA protein participates in the active expulsion of the As(III) from the cell and it is part of the *ars* operon, which has been proposed as the main mechanism used by the bacteria belonging to the *Exiguobacterium* genus to tolerate the As-associated stress. Interestingly, to date, this protein has never been described in a proteomic study (Belfiore et al., 2013; Sacheti et al., 2014). Cdr, and LuxS proteins, which are related to stress (Boylan et al., 2006; Sewald et al., 2007; Gohara and Yap, 2018), are up-regulated in the presence of both As species, suggesting a relevant role in the As tolerance. ArsA and Cdr proteins are the most up-regulated proteins in response to both As conditions, although the latter does not show significant changes in the SH1S21 strain. In an opposite way PunA, A0A1G1SP02 and Fur were the only proteins mainly down-regulated under both arsenic conditions for SH31 strain. Furthermore, those three proteins were not detected in SH1S21 strain, except for Fur which was detected and down-regulated in SH0S7 strains for both As conditions. It is interesting to note that the Cdr, LuxS and Fur proteins, all associated with stress response change their expression patterns mostly in SH31 and SH0S27 strains.

In regards to correlation between transcript and protein levels, a few cases are inconsistent and some hypothesis can be advanced. For example, Hpf protein in the SH31 strain could be post-transcriptionally regulated. The TpiA protein expression varies in a manner opposite to the level of expression of the transcript whatever the studied strain. As has been reported, correlation levels are highly variable and it has been suggested that this may be dependent on different biological factors, the efficiency of transduction, the half-life of the molecules and the technology used to generate the data (de Sousa Abreu et al., 2009; Maier et al., 2011).

## Conclusion

Bacteria from the *Exiguobacterium* genus are adapted to use multiple strategies that enable them to prosper under stress conditions that they face in their particular niches, specifically, the high toxicity generated by As. Mainly, those are based on the expulsion of the toxic compound, the maintenance of the redox state to avoid the oxidative stress and the repair of the unavoidable damages. They manage to do this using a great arsenal of proteins related to the protein synthesis, detoxification, generation of energy, transport and global stress. It is likely that the resistance/tolerance or response that a microorganism presents during stress conditions is originated by evolutionary adaptation to their ecologic niche. As it is observed in these three strains, they modify or supplement their basal defense mechanisms particularly against As to ensure their survival, as consequence they had different response patterns and tolerance levels. Only one protein, ArsA was regulated in the same way in the three strains independently of arsenic conditions suggesting that each strain sets up different resistance mechanisms/pathway to arsenic. This phenomenon can be further studied and explored to clarify the complex response net and molecular response to As-associated stress. Finally, we believe that the future challenges that should be embark are the generation of data bases and integration of computational/correlation methods for omics data from diverse sources with robust statistical analyses. Thus, we would be able to obtain the most of this large amount of data generated and ensure the interpretations and conclusion drawn from this are reliable, necessary for the next steps such as synthetic biology that depends on the availability of a large volume of high-quality data.

## Data Availability

All the proteomics data for this study has been deposited in the ProteomeXchange Consortium via the PRIDE partner repository with the dataset identifier PXD014701.

## Author Contributions

JC-S, CS, FM, and LM conceived and designed the study. JC-S, FM, CS, and FR performed the field work. JC-S and LM processed the samples and performed the 2D gels experiments. MS and MD performed the mass spectrometry. JC-S, NS, YS, VG, CP-E, EC-N, and LM analyzed the data. CS, FR, EC-N, FM, and LM contributed reagents, materials, and analysis tools. JC-S, CP-E, FR, EC-N, LM, and CS wrote the manuscript. All authors read and approved the final manuscript.

## Conflict of Interest Statement

The authors declare that the research was conducted in the absence of any commercial or financial relationships that could be construed as a potential conflict of interest.

## References

[B1] Acosta O. Custodio E. (2008). Impactos ambientales de las extracciones de agua subterránea en el salar de Huasco (norte de Chile). *Bol. Geol. Min.* 119 33–50.

[B2] Albarracín V. H. Gärtner W. Farias M. E. (2016). Forged under the sun: life and art of extremophiles from Andean lakes. *Photochem. Photobiol.* 92 14–28. 10.1111/php.12555 26647770

[B3] Albarracín V. H. Kurth D. Ordoñez O. F. Belfiore C. Luccini E. Salum G. M. (2015). High-Up: a remote reservoir of microbial extremophiles in Central Andean wetlands. *Front. Microbiol.* 6:1404. 10.3389/fmicb.2015.01404 26733008 PMC4679917

[B4] Altschul S. Gish W. Miller W. Myers E. Lipman D. (1990). Basic local alignment search tool. *J. Mol. Biol.* 215 403–410. 10.1016/S0022-2836(05)80360-2 2231712

[B5] Álvarez-Ordóñez A. Fernández A. Bernardo A. López M. (2010). Acid tolerance in *Salmonella typhimurium* induced by culturing in the presence of organic acids at different growth temperatures. *J. Food Microbiol.* 27 44–49. 10.1016/j.fm.2009.07.015 19913691

[B6] Anderson C. Cook G. (2004). Isolation and characterization of arsenate-reducing bacteria from arsenic-contaminated sites in New Zealand. *Curr. Microbiol.* 48 341–347. 10.1007/s00284-003-4205-3 15060729

[B7] Andreasen R. Li Y. Rehman Y. Ahmed M. Meyer R. L. Sabri A. N. (2018). Prospective role of indigenous *Exiguobacterium profundum* PT 2 in arsenic biotransformation and biosorption by planktonic cultures and biofilms. *J. Appl. Microbiol.* 124 431–443. 10.1111/jam.13636 29130635

[B8] Andres J. Bertin P. (2016). The microbial genomics of arsenic. *FEMS Microbiol. Rev.* 40 299–322. 10.1093/femsre/fuv050 26790947

[B9] Baker-Austin C. Dopson M. Wexler M. Sawers R. G. Stemmler A. Rosen B. P. (2007). Extreme arsenic resistance by the acidophilic archaeon ‘*Ferroplasma acidarmanus*’ Fer1. *Extremophiles* 11 425–434. 10.1007/s00792-006-0052-z 17268768

[B10] Bauer S. Grossmann S. Vingron M. Robinson P. N. (2008). Ontologizer 2.0—a multifunctional tool for GO term enrichment analysis and data exploration. *Bioinformatics* 24 1650–1651. 10.1093/bioinformatics/btn250 18511468

[B11] Belfiore C. Ordonez O. F. Farías M. E. (2013). Proteomic approach of adaptive response to arsenic stress in *Exiguobacterium* sp. S17, an extremophile strain isolated from a high-altitude Andean Lake stromatolite. *Extremophiles* 17 421–431. 10.1007/s00792-013-0523-y 23525943

[B12] Bhattacharjee H. Rosen B. (2007). “Arsenic metabolism in prokaryotic and eukaryotic microbes,” in *Molecular Microbiology of Heavy Metals*, eds Nies D. H. Silver S. (Heidelberg: Springer Press), 371–406. 10.1007/7171_2006_086

[B13] Bissen M. Frimmel F. (2003). Arsenic—a review. Part II: oxidation of arsenic and its removal in water treatment. *Acta Hydrochim. Hydrobiol.* 31 97–107. 10.1002/aheh.200300485

[B14] Blanch H. W. Clark D. S. (1997). *Biochemical Engineering.* New York, NY: CRC press.

[B15] Boylan J. A. Hummel C. S. Benoit S. Garcia-Lara J. Treglown-Downey J. Crane E. J. III (2006). *Borrelia burgdorferi* bb0728 encodes a coenzyme A disulphide reductase whose function suggests a role in intracellular redox and the oxidative stress response. *Mol. Microbiol.* 59 475–486. 10.1111/j.1365-2958.2005.04963.x 16390443

[B16] Cai L. Liu G. Rensing C. Wang G. (2009). Genes involved in arsenic transformation and resistance associated with different levels of arsenic-contaminated soils. *BMC Microbiol.* 9:4. 10.1186/1471-2180-9-4 19128515 PMC2631446

[B17] Canfora L. Bacci G. Pinzari F. Papa G. L. Dazzi C. Benedetti A. (2014). Salinity and bacterial diversity: to what extent does the concentration of salt affect the bacterial community in a saline soil? *PLoS One* 9:e106662. 10.1371/journal.pone.0106662 25188357 PMC4154724

[B18] Cánovas D. Cases I. De Lorenzo V. (2003). Heavy metal tolerance and metal homeostasis in *Pseudomonas putida* as revealed by complete genome analysis. *Environ. Microbiol.* 5 1242–1256. 10.1111/j.1462-2920.2003.00463.x 14641571

[B19] Cárdenas J. P. Moya F. Covarrubias P. Shmaryahu A. Levicán G. Holmes D. S. (2012). Comparative genomics of the oxidative stress response in bioleaching microorganisms. *Hydrometallurgy* 127 162–167. 10.1016/j.hydromet.2012.07.014

[B20] Casiano-Colón A. I. D. A. Marquis R. E. (1988). Role of the arginine deiminase system in protecting oral bacteria and an enzymatic basis for acid tolerance. *Appl. Environ. Microbiol.* 54 1318–1324. 2843090 10.1128/aem.54.6.1318-1324.1988 PMC202656

[B21] Castro-Severyn J. Remonsellez F. Valenzuela S. L. Salinas C. Fortt J. Aguilar P. (2017). Comparative genomics analysis of a new *Exiguobacterium* strain from Salar de Huasco reveals a repertoire of stress-related genes and arsenic resistance. *Front. Microbiol.* 8:456. 10.3389/fmicb.2017.00456 28377753 PMC5360010

[B22] Ceylan S. Yilan G. Akbulut B. S. Poli A. Kazan D. (2012). Interplay of adaptive capabilities of *Halomonas* sp. *AAD*12 under salt stress. *J. Biosci. Bioeng.* 114 45–52. 10.1016/j.jbiosc.2012.02.030 22575437

[B23] Ciprandi A. Baraúna R. A. Santos A. V. Gonçalves E. C. Carepo M. S. P. Schneider M. P. C. (2012). Proteomic response to arsenic stress in *Chromobacterium violaceum*. *J. Integ. OMICS* 2 69–73.

[B24] Cleiss-Arnold J. Koechler S. Proux C. Fardeau M. L. Dillies M. A. Coppee J. Y. (2010). Temporal transcriptomic response during arsenic stress in *Herminiimonas arsenicoxydans*. *BMC Genomics* 11:709. 10.1186/1471-2164-11-709 21167028 PMC3022917

[B25] Collins M. Lund B. Farrow J. Schleifer K. (1983). Chemotaxonomic study of an alkalophilic bacterium, *Exiguobacterium aurantiacum* gen. nov., sp. nov. *J. Gen. Microbiol.* 129 2037–2042. 10.1099/00221287-129-7-2037

[B26] da Costa W. L. O. de Aragão Araújo C. L. Dias L. M. de Sousa Pereira L. C. Alves J. T. C. Araújo F. A. (2018). Functional annotation of hypothetical proteins from the *Exiguobacterium antarcticum* strain B7 reveals proteins involved in adaptation to extreme environments, including high arsenic resistance. *PLoS One* 13:e0198965. 10.1371/journal.pone.0198965 29940001 PMC6016940

[B27] Daware V. Kesavan S. Patil R. Natu A. Kumar A. Kulkarni M. (2012). Effects of arsenite stress on growth and proteome of *Klebsiella pneumoniae*. *J. Biotechnol.* 158 8–16. 10.1016/j.jbiotec.2011.12.013 22209886

[B28] De Gregori I. Fuentes E. Rojas M. Pinochet H. Potin-Gautier M. (2003). Monitoring of copper, arsenic and antimony levels in agricultural soils impacted and non-impacted by mining activities, from three regions in Chile. *J. Environ. Monit.* 5 287–295. 10.1039/b211469k 12729270

[B29] de Sousa Abreu R. Penalva L. O. Marcotte E. M. Vogel C. (2009). Global signatures of protein and mRNA expression levels. *Mol. Biosyst.* 5 1512–1526. 10.1039/b908315d 20023718 PMC4089977

[B30] Demergasso C. S. Guillermo C. D. Lorena E. G. Mur J. J. P. Pedrós-Alió C. (2007). Microbial precipitation of arsenic sulfides in Andean salt flats. *Geomicrobiol. J.* 24 111–123. 10.1080/01490450701266605

[B31] Deutsch E. W. Csordas A. Sun Z. Jarnuczak A. Perez-Riverol Y. Ternent T. (2016). The ProteomeXchange consortium in 2017: supporting the cultural change in proteomics public data deposition. *Nucleic Acids Res.* 45 D1100–D1106. 10.1093/nar/gkw936 27924013 PMC5210636

[B32] Dorador C. Vila I. Imhoff J. F. Witzel K. P. (2008). Cyanobacterial diversity in Salar de Huasco, a high altitude saline wetland in northern Chile: an example of geographical dispersion? *FEMS Microbiol. Ecol.* 64 419–432. 10.1111/j.1574-6941.2008.00483.x 18410357

[B33] Dorador C. Vila I. Remonsellez F. Imhoff J. F. Witzel K. P. (2010). Unique clusters of Archaea in Salar de Huasco, an athalassohaline evaporitic basin of the Chilean Altiplano. *FEMS Microbiol. Ecol.* 73 291–302. 10.1111/j.1574-6941.2010.00891.x 20491927

[B34] Elias M. Wellner A. Goldin-Azulay K. Chabriere E. Vorholt J. A. Erb T. J. (2012). The molecular basis of phosphate discrimination in arsenate-rich environments. *Nature* 491 134–137. 10.1038/nature11517 23034649

[B35] Ferreccio C. Sancha A. (2006). Arsenic exposure and its impact on health in Chile. *J. Health Popul. Nutr.* 24 164–175. 17195557

[B36] Flynn H. C. Mc Mahon V. Diaz G. C. Demergasso C. S. Corbisier P. Meharg A. A. (2002). Assessment of bioavailable arsenic and copper in soils and sediments from the Antofagasta region of northern Chile. *Sci. Total Environ.* 286 51–59. 10.1016/s0048-9697(01)00962-7 11886099

[B37] Follmann H. Brownson C. (2009). Darwin’s warm little pond revisited: from molecules to the origin of life. *Naturwissenschaftem* 96 1265–1292. 10.1007/s00114-009-0602-1 19760276

[B38] Frazer B. (2012). Cancer cluster in Chile linked to arsenic contamination. *World Rep.* 379:603 10.1016/s0140-6736(12)60253-0 22355811

[B39] Gentleman R. C. Carey V. J. Bates D. M. Bolstad B. Dettling M. Dudoit S. (2004). Bioconductor: open software development for computational biology and bioinformatics. *Genome Biol.* 5:80. 15461798 10.1186/gb-2004-5-10-r80 PMC545600

[B40] Gihring T. Druschel G. McCleskey R. Hamers R. Banfield J. (2001). Rapid arsenite oxidation by *Thermus aquaticus* and *Thermus thermophilus*: field and laboratory investigations. *Environ. Sci. Tech.* 35 3857–3862. 11642444 10.1021/es010816f

[B41] Gohara D. W. Yap M. N. F. (2018). Survival of the drowsiest: the hibernating 100S ribosome in bacterial stress management. *Curr. Gen.* 64 753–760. 10.1007/s00294-017-0796-2 29243175 PMC6060826

[B42] Goyer R. A. Clarkson T. W. (2001). “Toxic effects of metals,” in *Casarett and Doull’s Toxicology: the Basic Science of Poisons*, eds Curtis D. Amdur M. O. (New York, NY: McGraw-Hill), 811–867.

[B43] Gutknecht R. Beutler R. Garcia-Alles L. F. Baumann U. Erni B. (2001). The dihydroxyacetone kinase of *Escherichia coli* utilizes a phosphoprotein instead of ATP as phosphoryl donor. *EMBO J.* 20 2480–2486. 10.1093/emboj/20.10.2480 11350937 PMC125457

[B44] Han B. Runnells T. Zimbron J. Wickramasinghe R. (2002). Arsenic removal from drinking water by flocculation and microfiltration. *Desalination* 145 293–298. 10.1016/s0011-9164(02)00425-3

[B45] Han Y. Zhou D. Pang X. Zhang L. Song Y. Tong Z. (2005). Comparative transcriptome analysis of *Yersinia pestis* in response to hyperosmotic and high-salinity stress. *Res. J. Microbiol.* 156 403–415. 10.1016/j.resmic.2004.10.004 15808945

[B46] Hardie K. R. Heurlier K. (2008). Establishing bacterial communities by ‘word of mouth’: LuxS and autoinducer 2 in biofilm development. *Nat. Rev. Microbiol.* 6 635–643. 10.1038/nrmicro1916 18536728

[B47] Hassan H. M. Troxell B. (2013). Transcriptional regulation by Ferric Uptake Regulator (Fur) in pathogenic bacteria. *Front. Cell Infect. Microbiol.* 3:59. 10.3389/fcimb.2013.00059 24106689 PMC3788343

[B48] Hernández K. L. Yannicelli B. Olsen L. M. Dorador C. Menschel E. J. Molina V. (2016). Microbial activity response to solar radiation across contrasting environmental conditions in Salar de Huasco, Northern Chilean Altiplano. *Front. Microbiol.* 7:1857. 10.3389/fmicb.2016.01857 27920763 PMC5118629

[B49] Herrera V. Amaro A. Carrasco C. (2014). *Speciation of Arsenic in a Saline Aquatic Ecosystem in Northern Chile. One Century of Arsenicosis in Latin America (1914-2014).* Boca Raton, FL: CRC Press.

[B50] Herrera V. De Gregori I. Pinochet H. (2009). Assesment of trace elements and mobility of arsenic and manganese in lagoon sediments of the Huasco and Coposa salt flats, Chilean altiplano. *J. Chil. Chem. Soc.* 54 454–459.

[B51] Hrimpeng K. Prapagdee B. Banjerdkij P. Vattanaviboon P. Dubbs J. Mongkolsuk S. (2006). Challenging *Xanthomonas campestris* with low levels of arsenic mediates cross-protection against oxidant killing. *FEMS Microbiol. Lett.* 262 121–127. 10.1111/j.1574-6968.2006.00383.x 16907748

[B52] Jomova K. Jenisova Z. Feszterova M. Baros S. Liska J. Hudecova D. (2011). Arsenic: toxicity, oxidative stress and human disease. *J. Appl. Toxicol.* 31 95–107. 10.1002/jat.1649 21321970

[B53] Joyce M. A. Fraser M. E. Brownie E. R. James M. N. Bridger W. A. Wolodko W. T. (1999). Probing the nucleotide-binding site of *Escherichia coli* succinyl-CoA synthetase. *Biochemistry* 38 7273–7283. 10.1021/bi990527s 10353839

[B54] Kilstrup M. Hammer K. Ruhdal Jensen P. Martinussen J. (2005). Nucleotide metabolism and its control in lactic acid bacteria. *FEMS Microbiol. Rev.* 29 555–590. 10.1016/j.femsre.2005.04.006 15935511

[B55] Kimbrel J. A. Ballor N. Wu Y. W. David M. M. Hazen T. C. Simmons B. A. (2018). Microbial community structure and functional potential along a hypersaline gradient. *Front. Microbiol.* 9:1492. 10.3389/fmicb.2018.01492 30042744 PMC6048260

[B56] Kruger M. C. Bertin P. N. Heipieper H. J. Arsène-Ploetze F. (2013). Bacterial metabolism of environmental arsenic—mechanisms and biotechnological applications. *Appl. Microbiol. Biotechnol.* 97 3827–3841. 10.1007/s00253-013-4838-5 23546422

[B57] Lara J. González L. E. Ferrero M. Díaz G. C. Pedrós-Alió C. Demergasso C. (2012). Enrichment of arsenic transforming and resistant heterotrophic bacteria from sediments of two salt lakes in Northern Chile. *Extremophiles* 16 523–538. 10.1007/s00792-012-0452-1 22555750

[B58] Leigh J. A. Dodsworth J. A. (2007). Nitrogen regulation in bacteria and archaea. *Annu. Rev. Microbiol.* 61 349–377. 10.1146/annurev.micro.61.080706.093409 17506680

[B59] Liu S. Athar M. Lippai I. Waldren C. Hei T. (2001). Induction of oxyradicals by arsenic: implication for mechanism of genotoxicity. *Proc. Natl. Acad. Sci. U.S.A.* 98 1643–1648. 10.1073/pnas.031482998 11172004 PMC29310

[B60] Maier T. Schmidt A. Güell M. Kühner S. Gavin A. C. Aebersold R. (2011). Quantification of mRNA and protein and integration with protein turnover in a bacterium. *Mol. Syst. Biol.* 7:511. 10.1038/msb.2011.38 21772259 PMC3159969

[B61] Malasarn D. Keeffe J. Newman D. (2008). Characterization of the arsenate respiratory reductase from *Shewanella* sp. strain ANA-3. *J. Bacteriol.* 190 135–142. 10.1128/jb.01110-07 17951391 PMC2223751

[B62] Mandal B. K. Suzuki K. T. (2002). Arsenic round the world: a review. *Talanta* 58 201–235. 10.1016/s0039-9140(02)00268-0 18968746

[B63] Mateos L. M. Villadangos A. F. Alfonso G. Mourenza A. Marcos-Pascual L. Letek M. (2017). The arsenic detoxification system in corynebacteria: basis and application for bioremediation and redox control. *Adv. Appl. Microbiol.* 99 103–137. 10.1016/bs.aambs.2017.01.001 28438267

[B64] Mi H. Thomas P. (2009). “PANTHER pathway: an ontology-based pathway database coupled with data analysis tools,” in *Protein Networks and Pathway Analysis*, eds Nikolsky Y. Bryant J. (Totowa, NJ: Humana Press), 123–140. 10.1007/978-1-60761-175-2_7 PMC6608593 19597783

[B65] Movahedzadeh F. Rison S. C. G. Wheeler P. R. Kendall S. L. Larson T. J. Stoker N. G. (2004). The *Mycobacterium tuberculosis* Rv1099c gene encodes a GlpX-like class II fructose 1, 6-bisphosphatase. *Microbiology* 150 3499–3505. 10.1099/mic.0.27204-0 15470127

[B66] Nelson D. L. Cox M. M. (2008). *Lehninger**: Principles of Biochemistry*, eds Nelson D. L. Cox M. M. (New York, NY: WH Freeman and Company), 1328.

[B67] Nies D. H. Silver S. (1995). Ion efflux systems involved in bacterial metal resistances. *J. Ind. Microbiol.* 14 186–199. 10.1007/bf01569902 7766211

[B68] Nyström T. (2004). MicroReview: growth versus maintenance: a trade-off dictated by RNA polymerase availability and sigma factor competition? *Mol. Microbiol.* 54 855–862. 10.1111/j.1365-2958.2004.04342.x 15522072

[B69] Nyunoya H. Lusty C. J. (1983). The carB gene of *Escherichia coli*: a duplicated gene coding for the large subunit of carbamoyl-phosphate synthetase. *Proc. Natl. Acad. Sci. U.S.A.* 80 4629–4633. 10.1073/pnas.80.15.4629 6308632 PMC384097

[B70] Ohtsuka T. Yamaguchi N. Makino T. Sakurai K. Kimura K. Kudo K. (2013). Arsenic dissolution from Japanese paddy soil by a dissimilatory arsenate-reducing bacterium *Geobacter sp*. OR-1. *Environ. Sci. Technol.* 47 6263–6271. 10.1021/es400231x 23668621

[B71] Okamura-Ikeda K. Ohmura Y. Fujiwara K. Motokawa Y. (1993). Cloning and nucleotide sequence of the *gcv* operon encoding the *Escherichia coli* glycine-cleavage system. *Eur. J. Biochem.* 216 539–548. 10.1111/j.1432-1033.1993.tb18172.x 8375392

[B72] Ordoñez O. F. Flores M. Dib J. Paz A. Farias M. E. (2009). Extremophile culture collection from andean lakes: extreme pristine environments that host a wide diversity of microorganisms with tolerance to UV radiation. *Microb. Ecol.* 58 461–473. 10.1007/s00248-009-9527-7 19495855

[B73] Ordoñez O. F. Lanzarotti E. Kurth D. Gorriti M. F. Revale S. Cortez N. (2013). Draft genome sequence of the polyextremophilic *Exiguobacterium* sp. *strain S*17, isolated from hyperarsenic lakes in the Argentinian Puna. *Genome Announc.* 1:e0480-13. 10.1128/genomeA.00480-13 23887911 PMC3735063

[B74] Ordoñez O. F. Lanzarotti E. O. Kurth D. G. Cortez N. Farias M. E. Turjanski A. G. (2015). Genome comparison of two *Exiguobacterium* strains from high altitude andean lakes with different arsenic resistance: identification and 3D modeling of the Acr3 efflux pump. *Front. Environ. Sci.* 3:50 10.3389/fenvs.2015.00050

[B75] Pacyna J. Pacyna E. (2001). An assessment of global and regional emissions of trace metals to the atmosphere from anthropogenic sources worldwide. *Environ. Rev.* 9 269–298. 10.1139/a01-012

[B76] Pandey N. Bhatt R. (2015). Arsenic resistance and accumulation by two bacteria isolated from a natural arsenic contaminated site. *J. Basic Microbiol.* 55 1275–1286. 10.1002/jobm.201400723 26095615

[B77] Pandey S. Rai R. Rai L. C. (2012). Proteomics combines morphological, physiological and biochemical attributes to unravel the survival strategy of *Anabaena* sp. *PCC*7120 under arsenic stress. *J. Proteom.* 75 921–937. 10.1016/j.jprot.2011.10.011 22057044

[B78] Perez-Riverol Y. Csordas A. Bai J. Bernal-Llinares M. Hewapathirana S. Kundu D. J. (2018). The PRIDE database and related tools and resources in 2019: improving support for quantification data. *Nucleic Acids Res.* 47 442–450. 10.1093/nar/gky1106 30395289 PMC6323896

[B79] Petänen T. Lyytikäinen M. Lappalainen J. Romantschuk M. Kukkonen J. V. K. (2003). Assessing sediment toxicity and arsenite concentration with bacterial and traditional methods. *Environ. Pollut.* 122 407–415. 10.1016/s0269-7491(02)00307-x 12547530

[B80] Pfaffl M. W. (2001). A new mathematical model for relative quantification in real-time RT–PCR. *Nucleic Acids Res.* 29:45. 10.1093/nar/29.9.e45 11328886 PMC55695

[B81] Philips S. Taylor M. (1976). Oxidation of arsenite to arsenate by *Alcaligenes faecalis*. *Appl. Environ. Microbiol.* 32 392–399. 10837 10.1128/aem.32.3.392-399.1976 PMC170076

[B82] Poirel J. Joulian C. Leyval C. Billard P. (2013). Arsenite-induced changes in abundance and expression of arsenite transporter and arsenite oxidase genes of a soil microbial community. *Res. J. Microbiol.* 164 457–465. 10.1016/j.resmic.2013.01.012 23396038

[B83] Qin J. Rosen B. Zhang Y. Wang G. Franke S. Rensing C. (2006). Arsenic detoxification and evolution of trimethylarsine gas by a microbial arsenite S-adenosylmethionine methyltransferase. *Proc. Natl. Acad. Sci. U.S.A.* 103 2075–2080. 10.1073/pnas.0506836103 16452170 PMC1413689

[B84] Remonsellez F. Castro-Severyn J. Aguilar Espinosa P. M. Fortt J. Salinas C. Barahona S. (2018). Characterization and salt response in recurrent halotolerant *Exiguobacterium* sp. *SH*31 isolated from sediments of Salar de Huasco, Chilean Altiplano. *Front. Microbiol.* 9:2228. 10.3389/fmicb.2018.02228 30294311 PMC6158405

[B85] Risacher F. Fritz B. (2009). Origin of salts and brine evolution of Bolivian and Chilean salars. *Aquat. Geochem.* 15 123–157. 10.1007/s10498-008-9056-x

[B86] Ritchie M. E. Phipson B. Wu D. Hu Y. Law C. W. Shi W. (2015). limma powers differential expression analyses for RNA-sequencing and microarray studies. *Nucleic Acids Res.* 43:e47. 10.1093/nar/gkv007 25605792 PMC4402510

[B87] Roh T. Steinmaus C. Marshall G. Ferreccio C. Liaw J. Smith A. H. (2018). Age at exposure to arsenic in water and mortality 30–40 years after exposure cessation. *Am. J. Epidemiol.* 187 2297–2305. 10.1093/aje/kwy159 30084889 PMC6211243

[B88] Sacheti P. Patil R. Dube A. Bhonsle H. Thombre D. Marathe S. (2014). Proteomics of arsenic stress in the gram-positive organism *Exiguobacterium* sp. *PS NCIM* 5463. *Appl. Microbiol. Biotechnol.* 98 6761–6773. 10.1007/s00253-014-5873-6 24931308

[B89] Schneider C. A. Rasband W. S. Eliceiri K. W. (2012). NIH Image to ImageJ: 25 years of image analysis. *Nat. Methods* 9 671–675. 10.1038/nmeth.2089 22930834 PMC5554542

[B90] Schnell R. Sriram D. Schneider G. (2015). Pyridoxal-phosphate dependent mycobacterial cysteine synthases: structure, mechanism and potential as drug targets. *Biochim. Biophys. Acta* 1854 1175–1183. 10.1016/j.bbapap.2014.11.010 25484279

[B91] Sewald X. Saum S. H. Palm P. Pfeiffer F. Oesterhelt D. Müller V. (2007). Autoinducer-2-producing protein LuxS, a novel salt-and chloride-induced protein in the moderately halophilic bacterium *Halobacillus halophilus*. *Appl. Environ. Microbiol.* 73 371–379. 10.1128/aem.01625-06 17085700 PMC1796989

[B92] Shahid F. Rizwan S. Khan M. W. Khan S. A. Naqshbandi A. Yusufi A. N. K. (2014). Studies on the effect of sodium arsenate on the enzymes of carbohydrate metabolism, brush border membrane, and oxidative stress in the rat kidney. *Environ. Toxicol. Pharmacol.* 37 592–599. 10.1016/j.etap.2014.01.012 24562057

[B93] Shannon P. Markiel A. Ozier O. Baliga N. S. Wang J. T. Ramage D. (2003). Cytoscape: a software environment for integrated models of biomolecular interaction networks. *Genome Res.* 13 2498–2504. 10.1101/gr.1239303 14597658 PMC403769

[B94] Slyemi D. Bonnefoy V. (2012). How prokaryotes deal with arsenic. *Environ. Microbiol. Rep.* 4 571–586. 10.1111/j.1758-2229.2011.00300.x 23760928

[B95] Smith A. Goycolea M. Haque R. Biggs M. (1998). Marked increase in bladder and lung cancer mortality in a region of Northern Chile due to arsenic in drinking water. *Am. J. Epidemiol.* 147 660–669. 10.1093/oxfordjournals.aje.a009507 9554605

[B96] Srivastava S. Verma P. C. Singh A. Mishra M. Singh N. Sharma N. (2012). Isolation and characterization of *Staphylococcus* sp. *strain NBRIEAG-*8 from arsenic contaminated site of West Bengal. *Appl. Microbiol. Biotechnol.* 95 1275–1291. 10.1007/s00253-012-3976-5 22410743

[B97] Sunita M. S. L. Prashant S. Chari P. B. Rao S. N. Balaravi P. Kishor P. K. (2012). Molecular identification of arsenic-resistant estuarine bacteria and characterization of their *ars* genotype. *Ecotoxicology* 21 202–212. 10.1007/s10646-011-0779-x 21879358

[B98] Takebe F. Hara I. Matsuyama H. Yumoto I. (2007). Effect of H2O2 under low- and high-aeration-level conditions on growth and catalase activity in *Exiguobacterium oxidotolerans* T-2-2T. *J. Biosci. Bioeng.* 104 464–469. 10.1263/jbb.104.464 18215632

[B99] Tapia J. Davenport J. Townley B. Dorador C. Schneider B. Tolorza V. (2018). Sources, enrichment, and redistribution of As, Cd, Cu, Li, Mo, and Sb in the Northern Atacama Region, Chile: implications for arid watersheds affected by mining. *J. Geochem. Explor.* 185 33–51. 10.1016/j.gexplo.2017.10.021

[B100] Tomoyasu T. Tabata A. Hiroshima R. Imaki H. Masuda S. Whiley R. A. (2010). Role of catabolite control protein A in the regulation of intermedilysin production by *Streptococcus intermedius*. *Infect. Immun.* 78 4012–4021. 10.1128/IAI.00113-10 20624907 PMC2937470

[B101] Trujillo C. Blumenthal A. Marrero J. Rhee K. Y. Schnappinger D. Ehrt S. (2014). Triosephosphate isomerase is dispensable *in vitro* yet essential for *Mycobacterium tuberculosis* to establish infection. *mBio* 5 e00085–14. 10.1128/mBio.00085-14 24757211 PMC3994511

[B102] Uribe J. Muñoz J. F. Gironás J. Oyarzún R. Aguirre E. Aravena R. (2015). Assessing groundwater recharge in an Andean closed basin using isotopic characterization and a rainfall-runoff model: Salar del Huasco basin, Chile. *Hydrogeol. J.* 32 1535–1551. 10.1007/s10040-015-1300-z

[B103] Vishnivetskaya T. A. Kathariou S. (2005). Putative transposases conserved in *Exiguobacterium* isolates from ancient Siberian permafrost and from contemporary surface habitats. *Appl. Environ. Microbiol.* 71 6954–6962. 10.1128/AEM.71.11.6954-6962.2005 16269730 PMC1287632

[B104] Vishnivetskaya T. A. Kathariou S. Tiedje J. M. (2009). The *Exiguobacterium* genus: biodiversity and biogeography. *Extremophiles* 13 541–555. 10.1007/s00792-009-0243-5 19381755

[B105] Wei T. Simko V. (2017). *R package “corrplot”: Visualization of a correlation matrix. Version 0.84.* Available at: https://github.com/taiyun/corrplot

[B106] Weiss S. Carapito C. Cleiss J. Koechler S. Turlin E. Coppee J.-Y. (2009). Enhanced structural and functional genome elucidation of the arsenite-oxidizing strain *Herminiimonas arsenicoxydans* by proteomics data. *Biochimie* 91 192–203. 10.1016/j.biochi.2008.07.013 18852016

[B107] Wickham H. (2016). *ggplot2: Elegant Graphics for Data Analysis.* New York, NY: Springer-Verlag.

[B108] Xiao K. Li L. Ma L. Zhang S. Bao P. Zhang T. (2016). Metagenomic analysis revealed highly diverse microbial arsenic metabolism genes in paddy soils with low-arsenic contents. *Environ. Pollut.* 211 1–8. 10.1016/j.envpol.2015.12.023 26736050

[B109] Xie F. Li G. Wang Y. Zhang Y. Zhou L. Wang C. (2017). Pyridoxal phosphate synthases PdxS/PdxT are required for *Actinobacillus pleuropneumoniae* viability, stress tolerance and virulence. *PLoS One* 12:e0176374. 10.1371/journal.pone.0176374 28448619 PMC5407770

[B110] Zhang J. Cao T. Tang Z. Shen Q. Rosen B. P. Zhao F.-J. (2015). Arsenic methylation and volatilization by arsenite S-adenosylmethionine methyltransferase in *Pseudomonas alcaligenes* NBRC14159. *Appl. Environ. Microbiol.* 81 2852–2860. 10.1128/AEM.03804-14 25681184 PMC4375323

[B111] Zhang Y. Chen S. Hao X. Su J.-Q. Xue X. Yan Y. (2016). Transcriptomic analysis reveals adaptive responses of an *Enterobacteriaceae* strain LSJC7 to arsenic exposure. *Front. Microbiol.* 7:636. 10.3389/fmicb.2016.00636 27199962 PMC4852401

[B112] Zhang Y. Ma Y.-F. Qi S.-W. Meng B. Chaudhry M. T. Liu S.-Q. (2007). Responses to arsenate stress by *Comamonas* sp. strain CNB-1 at genetic and proteomic levels. *Microbiology* 153 3713–3721. 10.1099/mic.0.2007/011403-0 17975079

