# Supplementary figures and images for: Arsenic Response of Three Altiplanic Exiguobacterium Strains With Different Tolerance Levels Against the Metalloid Species: A Proteomics Study
Source: Front Microbiol. 2019 Sep 26;10:2161. doi: 10.3389/fmicb.2019.02161 (PMC6775490; doi:10.3389/fmicb.2019.02161)

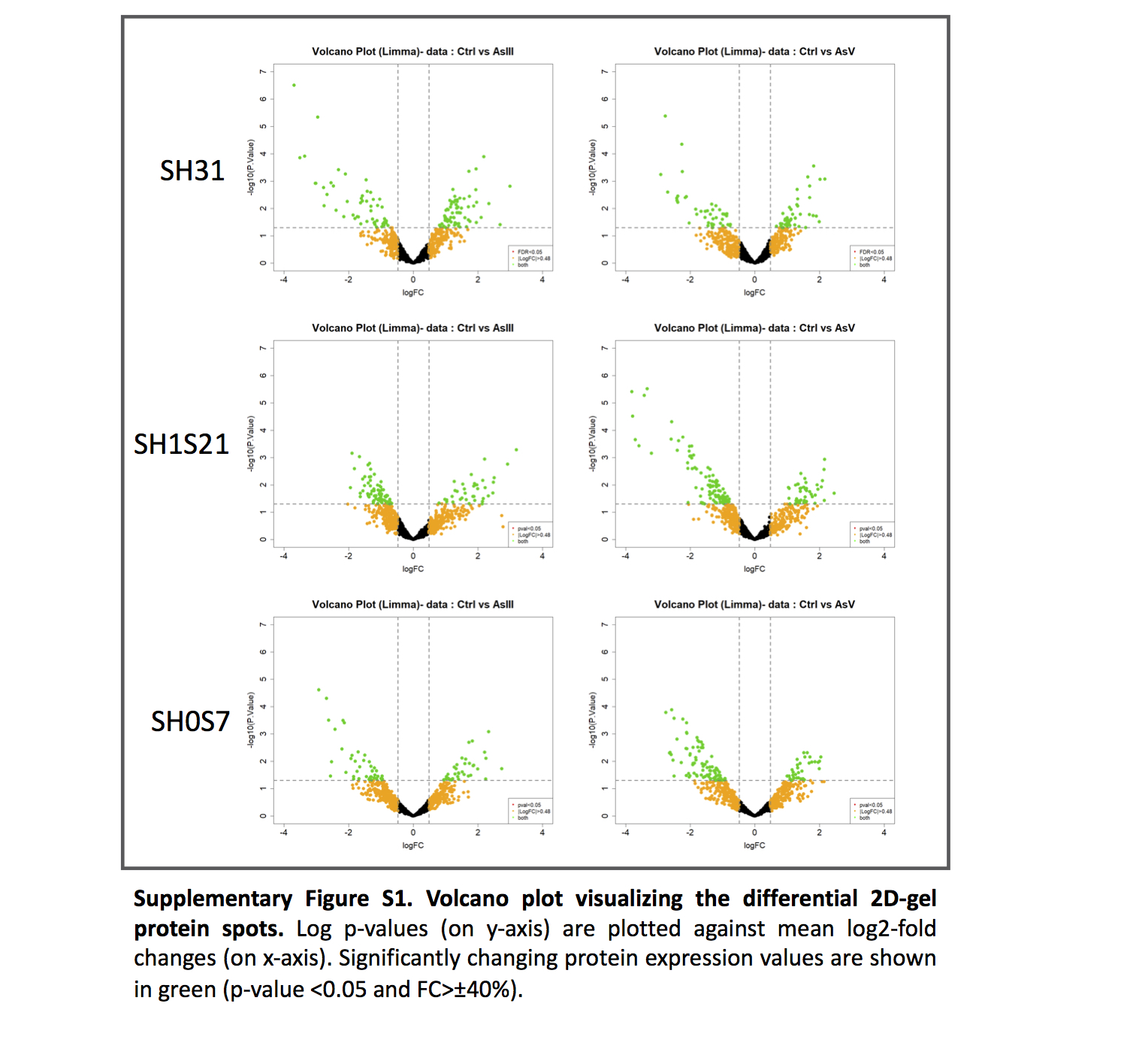

Supplement: Supplementary file 4 [file Image_1.JPEG]

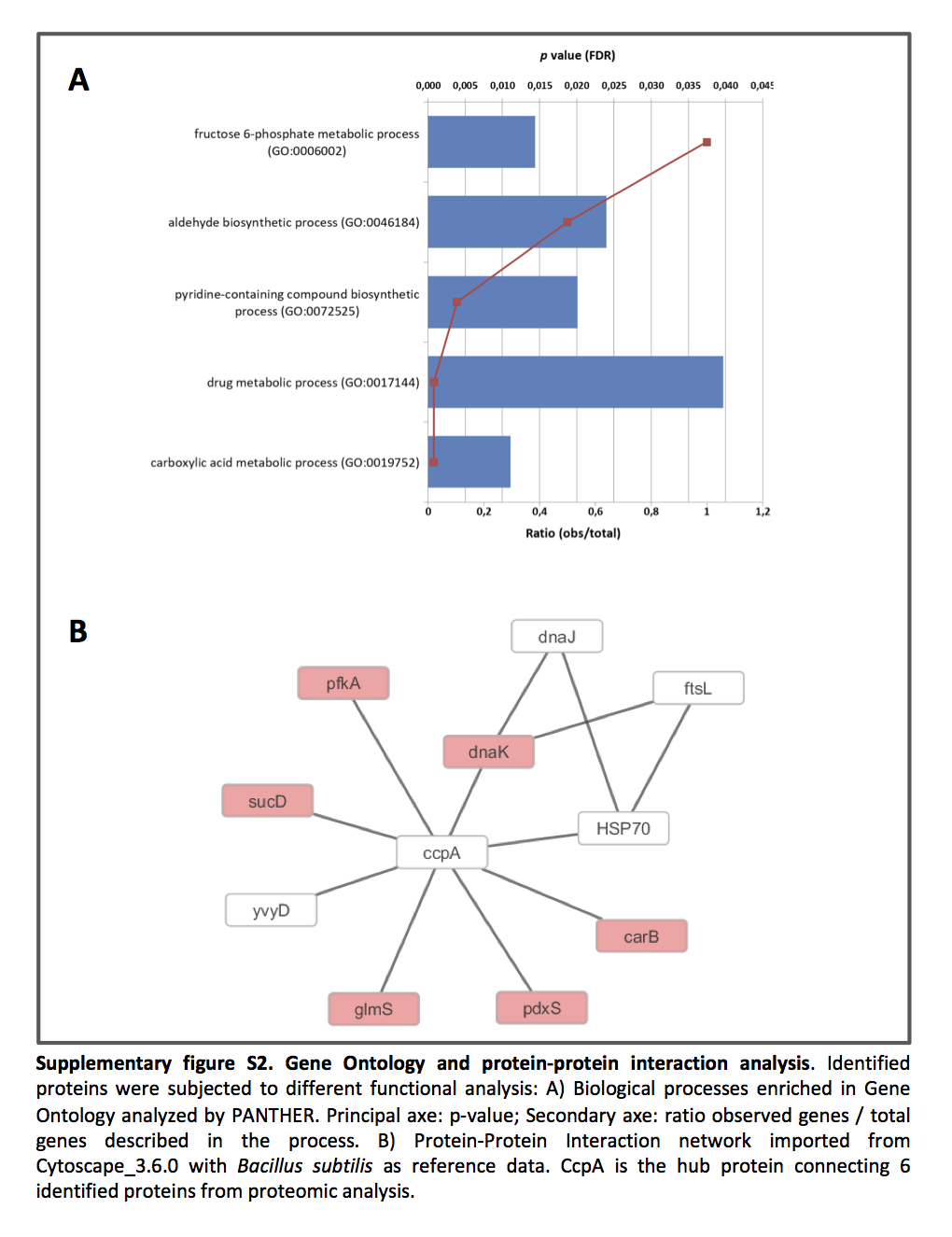

Supplement: Supplementary file 5 [file Image_2.JPEG]

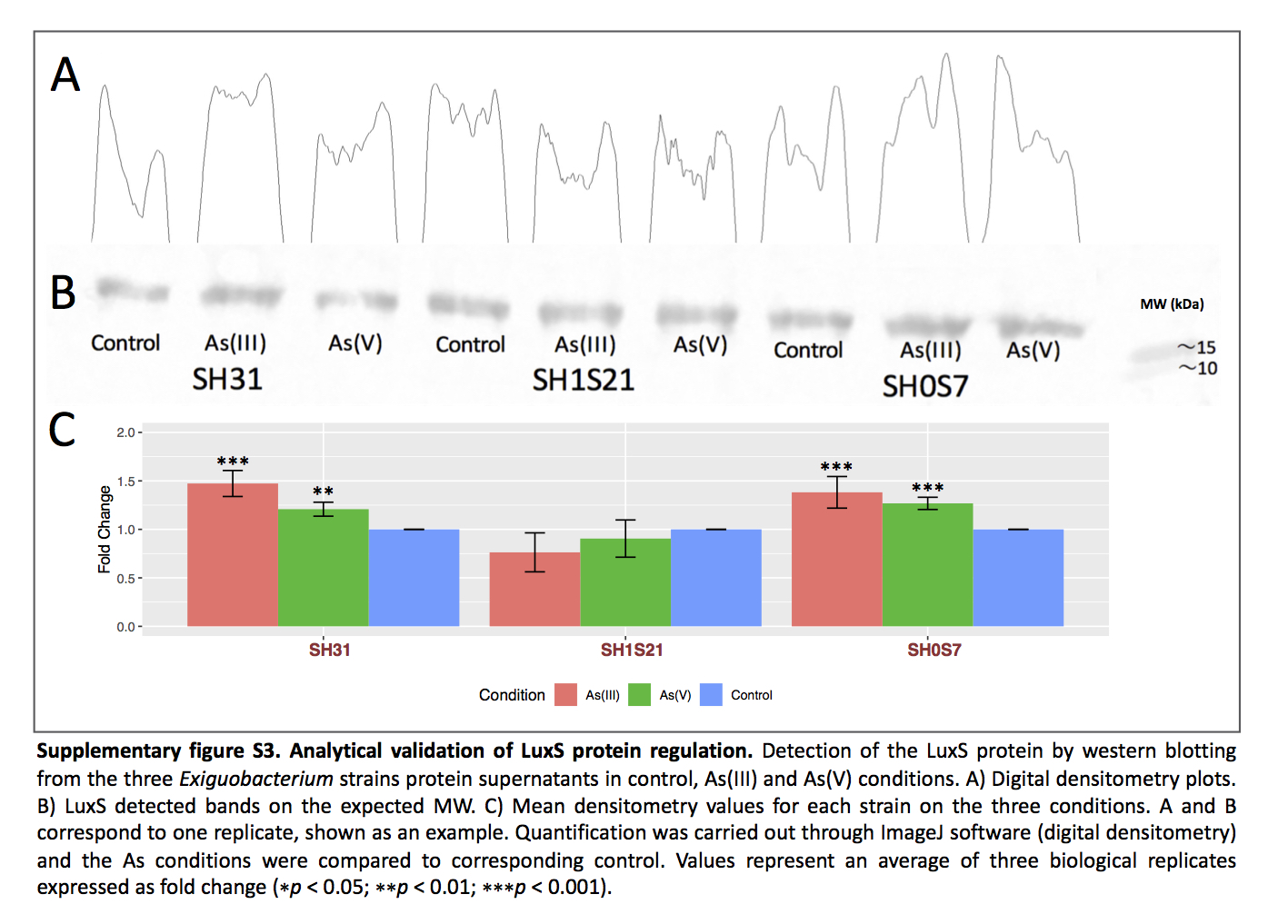

Supplement: Supplementary file 6 [file Image_3.JPEG]
